# Temperature Stress Resilience in Polar *Chlamydomonas* Is Regulated by Acclimation to Light and Salinity: Implications for Survival in a Changing World

**DOI:** 10.1111/ppl.71149

**Published:** 2026-10-14

**Authors:** Pomona Osmers, Allison Szenasi, Laura Kostyniuk, Nicholas Bradette, Marina Cvetkovska

**Affiliations:** ^1^ Department of Biology University of Ottawa Ottawa Ontario Canada

## Abstract

Aquatic algae are key primary producers in the Arctic and Antarctic, yet how cold‐water species respond to environmental change is poorly understood. The polar regions are increasingly exposed to frequent heat waves, leading to declining ice cover, increasing light availability, and decreasing salinity in polar waters. We compared three phylogenetically related but geographically distant polar *Chlamydomonas* species to test how habitat history shapes algal responses to light, salinity, and temperature stress. We assessed the growth, morphology, and photochemistry of psychrophilic *Chlamydomonas* acclimated to native‐like (lower light, higher salinity) and climate‐shifted (higher light, lower salinity) conditions. Next, we exposed acclimated cultures to heat shock and observed how acclimation affects algal temperature stress resilience. All three species acclimated to climate‐shifted conditions grew rapidly but showed the greatest sensitivity to temperature stress, with a rapid loss of viability and photosynthetic efficiency. In contrast, slow‐growing cultures acclimated to native‐like conditions exhibited significantly greater resilience to temperature stress. Our work is the first to directly link light and salinity acclimation with temperature resilience in psychrophilic algae, suggesting that fast‐growing polar green algae may be particularly vulnerable to increasingly frequent heat waves, with major implications for primary productivity in polar environments.

## 1 Introduction

The Arctic and Antarctic are the largest ecosystems on our planet, but the global cryosphere is shrinking. Air temperatures in the polar regions have increased 2–4× more than the global average, resulting in the loss of > 28 trillion tonnes of ice since 1994 (Slater et al. 2021). Modeling studies predict an alarming possibility of an ice‐free Arctic Ocean within the 21st century (Stroeve and Notz 2018; Wunderling et al. 2020), and there are reports of increased frequency and length of heatwaves, even in the interior of the Antarctic continent (e.g., 18.3°C; Turner et al. 2021; Feron et al. 2021). Primary production in the Polar Regions is largely dependent on photosynthetic microbes, including eukaryotic algae from all major lineages (Anderson and Macdonald 2015; Bax et al. 2021), many of which are obligate cold extremophiles (psychrophiles) that thrive in the cold (0°C–5°C) but cannot withstand even modest temperature increases (Morita 1975; Cvetkovska et al. 2017). Temperature sensitivity and loss of ice habitats make psychrophiles particularly threatened by current climate trends.

Light and salinity are key drivers in polar ecosystem dynamics and are particularly important for the growth of photosynthetic algae. Thinner ice and longer periods of seasonally open waters increase light availability, which supports higher rates of photosynthesis in aquatic habitats (Obryk et al. 2019; Castellani et al. 2022). Greater solar penetration within the water column also contributes to warming and increased melting at the ice‐water interface below the surface, resulting in runaway ice‐cover disintegration (Bers et al. 2013; Cárdenas et al. 2018; Lehnherr et al. 2018; Sherwell et al. 2022). The resulting rapid influx of meltwater leads to global “freshening” of marine waters, an effect that is particularly pronounced in the polar regions (Durack and Wijffels 2010; Bronselaer et al. 2018; Silvano et al. 2018). These environmental changes are accompanied by well‐documented ecosystem‐scale increases of primary production and greening of both poles (Lehnherr et al. 2018; Myers‐Smith et al. 2020; Williamson et al. 2020; Gray et al. 2020; Dalpadado et al. 2020; Roland et al. 2024). Despite this apparent positive effect on algal growth, little is known about the species composition of these microbial assemblages and the fate of obligate psychrophiles within them.

The order Chlamydomonadales (Chlorophyta) harbors more than a third of all confirmed algal psychrophiles, with a wide distribution in Antarctic, Arctic, and alpine habitats (Cvetkovska et al. 2017). This group includes several well‐described green algae with sequenced genomes that have been elevated to the status of models for photosynthetic life at the extreme (Cvetkovska et al. 2019; Zhang et al. 2019, 2020, 2021). Among these, the Antarctic *Chlamydomonas priscui* (formerly UWO241; Morgan‐Kiss and Guiry 2024) is the only psychrophile that has been studied in the context of temperature stress. Exposure to acute non‐permissive temperature (24°C) results in alterations in global metabolomic and transcriptomic responses after 6 h (Cvetkovska, Zhang, et al. 2022), followed by a rapid drop in photosynthetic rates after 12 h and cellular death with prolonged exposure (Possmayer et al. 2011). These studies suggest that psychrophilic algae have a limited ability to induce a heat stress response (HSR) similar to that of their mesophilic relatives (Schroda et al. 2015), which is insufficient in overcoming their inherent temperature sensitivity.

These breakthrough studies opened the door to understanding psychrophilic responses to environmental stress, but *C. priscui* is unique even among polar Chlamydomonadales. This alga is endemic to the deep photic zone of the Antarctic Lake Bonney in the McMurdo Dry Valleys (Neale and Priscu 1995), where the perennial 3–6 m thick ice cover severely restricts external inputs, resulting in a highly stable and stratified environment characterized by strict temperature, light, salinity, and nutrient profiles (Patriarche et al. 2021). In its native environment, *C. priscui* experiences permanently low temperatures (~4°C), very low light (~10 μmol m^−2^ s^−1^) biased in blue‐green wavelengths (400–500 nm), high salinity (~700 nm NaCl), high O_2_ saturation (30–40 mg L^−1^) and low nutrient levels (Neale and Priscu 1995). It has been postulated that life under such stable conditions has resulted in a “locked” physiology that confers adaptive advantages in an extreme and stable habitat, but lacks the capacity to respond to short‐term environmental challenges (recently reviewed in Cvetkovska, Vakulenko, et al. 2022; Hüner et al. 2022, 2024). Therefore, while *C. priscui* is undoubtedly the best characterized polar green alga, its responses to environmental stress may not be representative of psychrophiles in general.

Psychrophilic *Chlamydomonas* have been detected in virtually all polar habitats (Cvetkovska et al. 2017), but their stress resilience capabilities are largely unknown. Open marine waters, sea ice, and snowfields are populated by a wide diversity of photosynthetic microbes (Balzano et al. 2012; van Leeuwe et al. 2018; Davey et al. 2019; Gérikas Ribeiro et al. 2020; Dorrell et al. 2023) that experience annual freeze–thaw cycles accompanied by dramatic light, salinity, and temperature fluctuations (Tremblay et al. 2012; Urbański and Litwicka 2022; Spolaor et al. 2024). This natural seasonal variability might influence the ability of psychrophilic algae to acclimate to new conditions, and we hypothesized that polar algae originating from a dynamic environment will have an increased ability to withstand environmental stress.

To address this, we compared the physiology of three phylogenetically related but geographically distant psychrophilic *Chlamydomonas* species in the face of acute and sustained environmental challenge. In addition to *C. priscui*, we focused on two of its relatives: (1) the euryhaline *Chlamydomonas malina* native to the open waters of the Arctic Beaufort Sea that experience seasonal freeze–thaw cycles, deeper light penetration, and frequent vertical mixing (Taylor et al. 2013); and (2) the freshwater *Chlamydomonas klinobasis* isolated from ephemeral snowfields on Spitsbergen Island in the Arctic (Hulatt et al. 2017), a dynamic oligotrophic environment characterized by high light intensities on the surface and rapid light attenuation within the snow pack (Stibal et al. 2007). Using light and salinity as variables, we characterized the physiological acclimation strategies of these species to climate‐shifted steady‐state conditions and their responses to subsequent temperature stress. This work provides a unique opportunity to compare the stress resilience of closely related species that originate from cold but otherwise very different environments and enhances our understanding of climate‐sensitive psychrophilic biology.

## 2 Materials and Methods

### 2.1 Algae Cultivation and Experiment Design

*Chlamydomonas priscui* (formerly UWO241; CCMP1619, https://ncma.bigelow.org/) was isolated from 17 m below the ice surface in Lake Bonney, Antarctica (Neale and Priscu 1995). *Chlamydomonas malina* (RCC2488; https://roscoff‐culture‐collection.org/) was isolated from the surface waters of the Beauford Sea in the Canadian Arctic (Balzano et al. 2012). *Chlamydomonas klinobasis* (CCCRyo 050‐99; https://cccryo.fraunhofer.de) originates from a snowfield on Spitsbergen in the Svalbard archipelago (Hulatt et al. 2017).

Axenic stocks were maintained on Bold's Basal Media (BBM) agar slants at 4°C with continuous illumination (~50 μmol m^−2^ s^−1^). Working liquid stocks were seeded from agar‐grown cultures in 250 mL Erlenmeyer flasks in BBM media. Experimental cultures were grown in custom‐made closed culture bioreactors suspended in temperature‐regulated aquaria and aerated with sterile ambient air provided by aquarium pumps. Continuous light (10 μmol m^−2^ s^−1^ or 100 μmol m^−2^ s^−1^) was provided by full‐spectrum LED bulbs (FusionBright, T5 LED 18W). Intensity was modulated with neutral density filters and monitored with a quantum light sensor (Model LI‐189; Li‐COR). Salinity was modified by supplementing BBM media with NaCl at the appropriate concentration (10 and 700 mM for *C. priscui*; 10–600 mM for *C. malina* ; 0.43–70 mM for *C. klinobasis*). All cultures were allowed to acclimate to these conditions for at least 4 weeks before experimentation. For temperature stress experiments, algae were cultured at 4°C until the mid‐exponential growth phase (OD_750_ = 0.3–0.4). Stress was initiated by moving the cultures to a non‐permissive temperature: 24°C for *C. priscui* and 22°C for *C. klinobasis* and *C. malina* . All experiments were done on cultures during mid‐exponential growth and with at least three biological replicates (independently seeded cultures), each with four technical replicates (individual bioreactor tubes).

### 2.2 Phylogenetic Analysis

To determine the phylogenetic relationship between the three species, we constructed gene trees using the nuclear‐encoded 18S *rRNA* and plastid‐encoded *RBCL* genes. The sequenced genomes of *C. priscui* (Zhang et al. 2021) and *C. klinobasis* (JGI Project ID: 1106927; https://genome.jgi.doe.gov/portal/ChlkliStandDraft_3) were used to source the *RBCL* and 18S *rRNA* nucleotide sequences. To obtain *RBCL* sequences from *C. malina* , tissue was collected during the mid‐exponential phase and total DNA was isolated as previously described (Zhang et al. 2021). Gene fragments were amplified using universal green algae *RBCL* primers (Forward: 5′‐GGTACTTGGACAACAGTTTGGAC‐3′; Reverse: 5′‐TACGCAACCTCTCTGGCAAAG‐3) (Hadi et al. 2016), and Sanger sequenced (Génome Québec, Montreal, Canada). All other sequences were obtained from GenBank via BLASTn searches within the Chlamydomonadales (taxid: 3042), using sequences from model algae and the species studied here as queries.

We obtained all available sequences from the Monadinia and Moewusinia clades (sensu Nakada; Nakada et al. 2008) and from selected representatives from other Chlamydomonadales clades. The data were manually curated to remove duplicate and low‐quality sequences. To avoid overrepresentation, we removed sequences with ≥ 98% identity that originated from the same species but different strains. This resulted in a total of 64 *RBCL* and 111 18S *rRNA* sequences used in downstream analyzes. All sequences were aligned using MAFFT (Katoh and Standley 2013) and trimmed to remove gaps and ambiguously aligned regions with BMGE (Criscuolo and Gribaldo 2010). Phylogenetic relationships were inferred using maximum likelihood in PhyML v.3.0 (Guindon et al. 2010) via the NGPhylogeny platform (Lemoine et al. 2019). The best‐fitting model was selected using the Smart Model Selection (SMS) based on the Akaike Information Criterion (AIC) (Lefort et al. 2017). Tree topology searches were performed using the SPR algorithm, and statistical support for branches was evaluated using 500 non‐parametric bootstrap replicates. Trees were rooted at species belonging to the Sphaeropleales, a sister clade to the Chlamydomonadales (Buchheim et al. 2001; Lemieux et al. 2015), and annotated in iTOL (Letunic and Bork 2021).

### 2.3 Growth and Viability Measurements

Algal growth was monitored by measuring pigment content, cell counts, and optical density (OD). To measure pigment content, 1 mL of culture was pelleted by centrifugation (3 min, 16,000 g), the supernatant was removed and replaced with 90% (v/v) acetone. The pellet was re‐suspended, centrifuged again to remove cellular debris (3 min, 16,000 g), and the absorbance of the supernatant was measured spectroscopically at 467, 664, and 647 nm (Cary 60, Agilent Technologies). Chlorophyll (Chl) *a* and *b* content was calculated according to Jeffrey and Humphrey (1975). Carotenoid (Car) content was calculated according to Sun et al. (1998), with the absorbance maximum shifted from ß‐carotene to astaxanthin. Bleaching, or the loss of Chl during stress, was calculated as a percentage of the Chl content immediately before stress initiation. Cell numbers were quantified with an automatic cell counter (Countess II FL, ThermoFisher Scientific) using brightfield imaging (EVOS Light Cube, white) and Chl autofluorescence imaging (EVOS Cy5 Light Cube, Ex/Em = 628/685 nm). Change in OD was measured at 750 nm (Cary 60, Agilent Technologies). To avoid over‐ or underestimating growth rates due to changes in cellular morphology that may affect cell motility and sample heterogeneity, we mixed each sample thoroughly by inversion to resuspend any non‐motile sinking cells and ensured the absence of large cell aggregates that may interfere with OD measurements. All OD and pigment measurements were done in triplicate and averaged to reduce random variability from heterogeneous sampling. Growth rates were calculated using OD_750_ values during exponential growth.

Cell viability was quantified by labeling dead algal cells with the fluorescent dye SYTOX Green (Ex/Em = 504/523 nm; ThermoFisher Scientific). SYTOX Green, dissolved in DMSO, was added to algal samples at a final concentration of 2 μM, followed by 15 min incubation in the dark prior to fluorescent imaging. Green fluorescence, corresponding to dead cells, was measured with an automated cell counter (EVOS GFP Light Cube, Ex/Em = 470/525 nm). Cell death was quantified as a proportion of green fluorescing cells to the total number of cells.

### 2.4 Flow Cytometry and Microscopy

Cellular size and complexity were examined using a flow cytometer (Gallios, Beckman Coulter). In each case, SYTOX Blue (Ex/Em = 405/480 nm; ThermoFisher Scientific) dissolved in DMSO was added to algal culture (~1 × 10^6^ cells mL^−1^) at a final concentration of 1 μM and incubated in the dark for 5 min with gentle agitation at the experimental temperature. Data were collected as at least 20,000 events for each sample, at a flow rate of 500 events s^−1^. Algal cells were identified through Chl autofluorescence using the FL7 detector (725 ± 20 nm), thus eliminating cellular debris or contaminants from downstream analyzes. Dead cells stained with SYTOX Blue were detected by the FL9 detector (450 ± 40 nm) and excluded from further analyzes.

Data was analyzed using the Kaluza Software (Beckman Coulter, v2.1). Cell size and granularity were obtained from the forward scatter (FS) and side scatter (SSC) measurements, respectively. The ratio between FS height and FS area was used to determine cell size and estimate the proportion of single cells and palmelloids. These measurements were further confirmed by measuring FS and SSC to estimate internal complexity, expected to be higher in multi‐cell palmelloid colonies. The cellular morphology was further confirmed by the presence of two distinct chlorophyll autofluorescence peaks exhibiting minimal overlap, as demonstrated previously (Szyszka‐Mroz et al. 2022). Algae were visualized using brightfield imaging on a Zeiss AxioImager microscope using the Zen Pro Blue V. 3.2.0 software (Carl Zeiss AG).

### 2.5 Room Temperature Chlorophyll a Fluorescence

In vivo room temperature Chl *a* fluorescence was measured using a pulse amplitude modulated chlorophyll fluorometer (DUAL‐PAM‐100; Walz) with the WinControl software. Live cultures (1 mL; 2 μg Chl mL^−1^) were stirred with a magnetic stir bar and kept at constant temperature (4°C or 22°C–24°C) using a water‐jacketed quartz cuvette connected to a circulating water bath. After 10 min of dark adaptation, Chl fluorescence at open PSII reaction centers (*F*_*o*_) was excited by a non‐actinic measuring light (0.34 μmol m^−2^ s^−1^) pulsed at 20 Hz. Maximum fluorescence at closed PSII reaction centers in the dark (*F*_*m*_) was induced by a saturating pulse (200 ms, 10,286 μmol m^−2^ s^−1^). To measure maximal fluorescence at closed PSII reactions centers (*F*_*m*_′) and the steady‐state fluorescence in the light (*F*), saturating flashes were applied in 30 s intervals under constant actinic light of 27 μmol m^−2^ s^−1^. All photosynthetic parameters were calculated according to Kramer et al. 2004. Maximum PSII photochemical efficiency (*F*_*V*_/*F*_*M*_) was calculated as *F*_*M*_–*F*_*o*_/*F*_*M*_, using dark‐adapted cells. The quantum yield of steady‐state photosynthesis in the light was calculated as: Y(II) = (*F*_*M*_′–*F*_*S*_)/*F*_*M*_′, Y(NPQ) = (*F*/*F*_*M*_′)−(*F*/*F*_*M*_), Y(NO) = *F*/*F*_*M*_, where Y(II) is the yield of PSII photochemistry, Y(NPQ) is the yield of non‐photochemical energy dissipation by down‐regulation through antenna quenching, and Y(NO) is the yield of all other processes involved in non‐photochemical energy losses. The relative electron transport rate (ETR) at PSII was determined as ETR = Y(II) × (PFD × 0.84) × 0.5.

### 2.6 Statistical Analysis

All statistical analyzes were conducted in R using the rstatix (Kassambara 2025), car (Fox and Weisberg 2019), and emmeans (Lenth and Piaskowski 2026) packages (www.r‐project.org). Growth rates, pigment quantification, and cell sizes were analyzed by two‐way ANOVA and Tukey's post hoc test, with salinity and light intensity as independent variables. Cellular viability was analyzed using linear models and Tukey's post hoc test, with salinity, light intensity, and time as independent variables. Photosynthetic measurements were analyzed by three‐way repeated measures ANOVA with Dunnett's post hoc test, using salinity and light intensity as independent variables.

## 3 Results

### 3.1 The Phylogeny and Morphology of Psychrophilic Chlamydomonas

The phylogenetic analysis of the *RBCL* and 18S *rRNA* sequences positioned all three psychrophilic *Chlamydomonas* species used in this work within the Monadinia and Moewusinia clades of the Clamydomonadales (Figures 1, S1 and S2). The topologies of the phylogenetic trees were consistent with previously published data (Nakada et al. 2008, 2016; Lemieux et al. 2015). The phylogenetic position of the Antarctic *C. priscui* in a unique lineage within the Moewusinia clade was previously described (Possmayer et al. 2016). Here, we report that its closest known relative is the Arctic *C. malina* , with high bootstrap support (99%; Figures 1, S1 and S2). These species shared a close phylogenetic relationship with *Chlamydomonas* sp. WAP05 and WAP30 isolated from the Southern Ocean (Soto et al. 2023). Our analysis provided strong support (87%–96%, Figures 1, S1 and S2) for the placement of the snow alga *C. klinobasis* in the same clade as *Microglena monadina* (Demchenko et al. 2012; Trentin et al. 2022) and other species from the polar regions (e.g., *Chlamydomonas* sp. ICE‐L, ICE‐W, ICE‐MDV, KNF0022) (Liu et al. 2006; Eddie et al. 2008; Jung et al. 2016; Cook et al. 2019).

**FIGURE 1:**
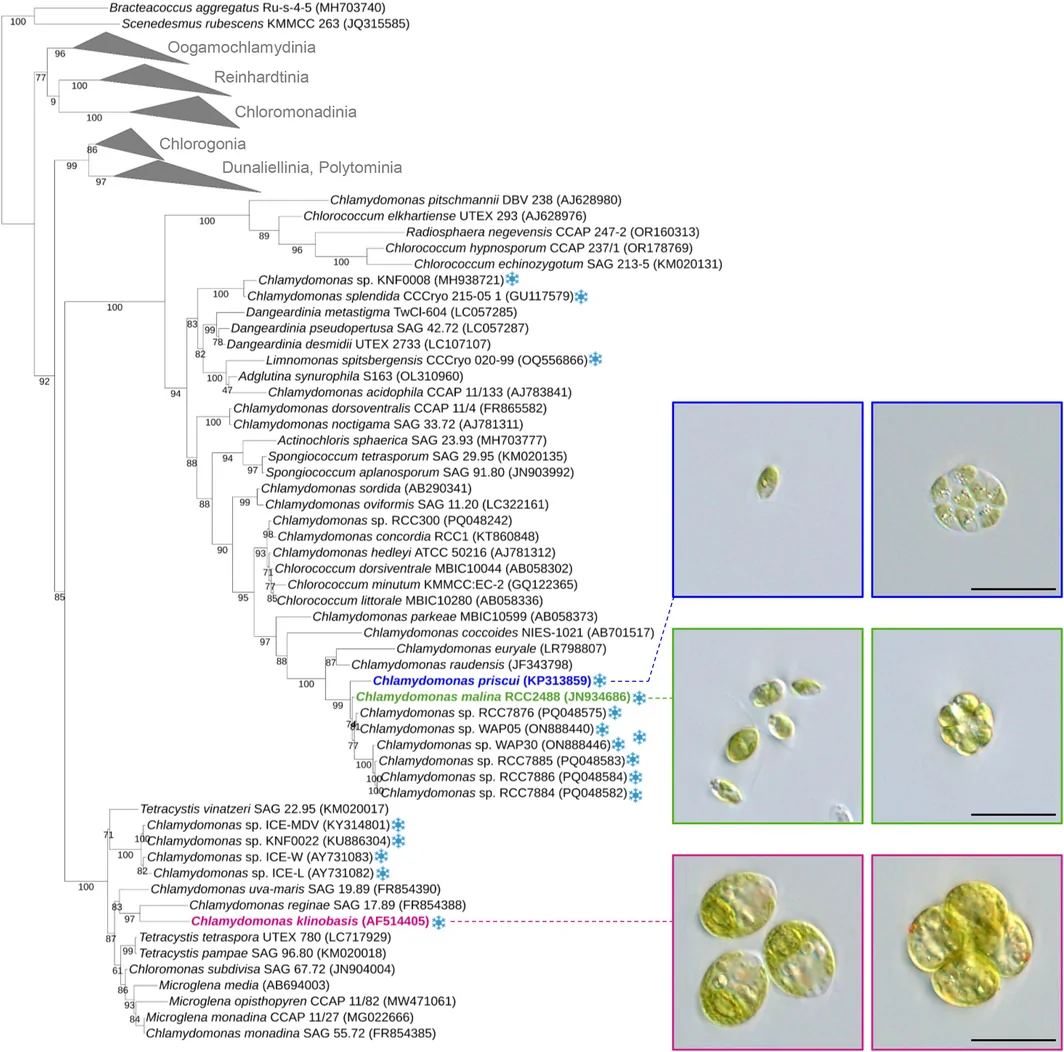
Phylogenetic tree of 111 18S rRNA nucleotide sequences inferred from using maximum likelihood analysis is shown to scale (substitutions per site), with branch values representing bootstrap support. GenBank accession numbers are provided in brackets beside the name of the organism. Collapsed clades contain select green algal species within major *Chlamydomonas* taxa and are represented by triangles at the branch names, while the Monadinia and Moewusinia clades are shown in full. Algae isolated from polar environments are indicated with a blue symbol. The species considered in this study are highlighted in blue (*Chlamydomonas priscui*), green (*Chlamydomonas malina*) and mauve (*Chlamydomonas klinobasis*). The inset shows light micrographs of these species as single cells (left) and palmelloids (right). Scale bar = 10 μm.

Morphologically, all three species exist as biflagellate free‐swimming single cells on non‐motile palmelloid colonies, where multiple single cells are encased in a shared extracellular matrix (Figure 1, inset). The occurrence of these morphological variants has been described in *C. priscui* (Pocock et al. 2004; Possmayer et al. 2016; Szyszka‐Mroz et al. 2022), and here we showed that single cells and palmelloids also co‐occur in *C. malina* and C. *klinobasis* populations. As previously reported (Pocock et al. 2004; Possmayer et al. 2016), *C. priscui* single cells were ellipsoidal or sub‐fusiform, approximately 6–7 μm long, with a single cup‐shaped chloroplast and a very small eyespot (Figure 1, inset). The morphology of *C. malina* was similar, with ovoid, ellipsoidal or sub‐fusiform cells with a single chloroplast, albeit smaller in size (~4–6 μm long) and displaying a more prominent eyespot compared to *C. priscui* (Figure 1, inset). Finally, single cells in the freshwater *C. klinobasis* were subspherical or ovoid and significantly larger (~12–13 μm in length) compared to the halotolerant species, with a very prominent eyespot visible in both single cells and palmelloids (Figure 1, inset).

### 3.2 Salinity and Light Affect the Growth and Physiology of Psychrophilic Chlamydomonas

The three psychrophilic *Chlamydomonas* species used here originate from geographically and environmentally distinct habitats. Thus, we monitored the growth and morphology of these algae at environmentally relevant (rather than identical) conditions (Table 1). For the halotolerant *C. priscui* and *C. malina* , the high salinity (HS) treatment was chosen to mimic their natural habitat (700 and 300 mM, respectively) (Smoot and Hopcroft 2017; Patriarche et al. 2021). For the freshwater *C. klinobasis*, the HS treatment (30 mM) was determined experimentally to represent the salinity that supports robust growth comparable to that of *C. priscui* and *C. malina* at high salinity (Figure S3a; Table S1). Conversely, the low salinity treatment for *C. klinobasis* (LS, 0.43 mM) was chosen as an approximation of salinities measured in snow (Tonboe et al. 2021; Saha et al. 2025). For the halotolerant species, low salinity was defined as the salinity at which these algae exhibited the highest growth rates comparable to those of *C. klinobasis*, previously reported as 10 mM for *C. priscui* (Possmayer et al. 2011; Cvetkovska, Zhang, et al. 2022) and experimentally determined as 30 mM for *C. malina* (Figure S3b; Table S1). For all three species (Table 1), the low light treatment (LL, 10 μmol m^−2^ s^−1^) represents the intensity received by polar algae in their natural habitat under ice or snow cover (Neale and Priscu 1995; Stibal et al. 2007; Hill et al. 2018). The high‐light treatment (HL, 100 μmol m^−2^ s^−1^) mimics light availability in shallow open waters (Hill et al. 2018; Sherwell et al. 2022) and is the recommended intensity for culturing various *Chlamydomonas* species in the lab (Hui et al. 2023). All three species had comparable growth rates when acclimated to these experimental conditions (Table S1).

**TABLE 1:** Algal species, their geographical location, and the experimental conditions used in this work.

Species	Geographical location and habitat	Salinity (mM NaCl)		Light (μmol m^−2^ s^−1^)	
		High (HS)	Low (LS)	High (HL)	Low (LL)
*C. priscui*	Antarctic, perennially ice‐covered Lake bonney	700*	10	100	10*
*C. malina*	Arctic, seasonally ice‐covered Beaufort Sea	300*	30	100	10
*C. klinobasis*	Arctic, ephemeral snow fields on Svalbard Island	30	0.43*	100	10

*Note:* The salinity and light intensity that resemble each species' natural habitat are emphasized with (*). While C. priscui originates from a stable environment characterized by low light availability, *C. malina* and *C. klinobasis* originate from dynamic environments and experience variable light conditions.

Light availability was a major driver of growth in all three species. In *C. malina* and *C. klinobasis*, growth was also dependent on salinity or the interaction between light and salinity (Figure 2a, Table S2). *C. priscui* cultures achieved maximal growth when acclimated to higher light intensity (0.35–0.49 ΔOD_750_ day^−1^) compared to those acclimated to low light (0.19–0.26 ΔOD_750_ day^−1^), regardless of salinity (Figure 2a). In contrast, *C. malina* and *C. klinobasis* cultures exhibited highest growth rates with higher light availability and lower salinity (0.71 and 0.47 ΔOD_750_ day^−1^, respectively), and decreased growth rates when cultured at higher salinities (0.45 and 0.28 ΔOD_750_ day^−1^, respectively). Both species grew significantly slower when cultured at lower light intensity, regardless of the salinity (0.21–0.23 ΔOD_750_ day^−1^ for *C. malina* ; 0.15–0.23 ΔOD_750_ day^−1^ for *C. klinobasis*) (Figure 2a).

**FIGURE 2:**
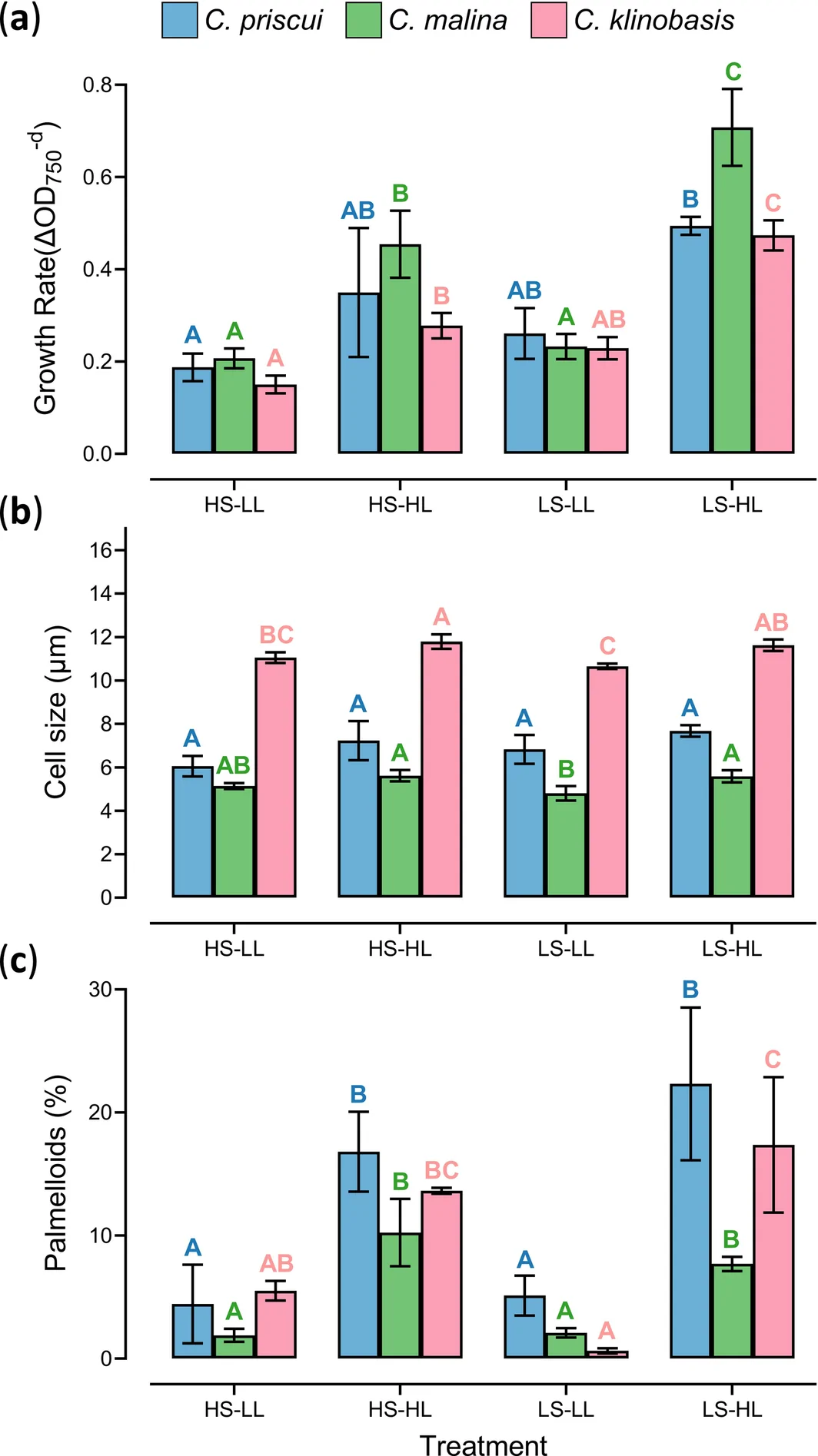
The effects of salinity and light on the growth and morphology of *C. priscuii*, *C. malina* and *C. klinobasis*. (a) Maximal growth rates. (b) Average cell size. (c) A percentage of cells present as palmelloid colonies. In all cases, algae were cultured at 4°C and acclimated to high salinity (HS), low salinity (LS), high light (HL) or low light (LL). Data are means ± SD of at least three biological replicates, each with at least four technical replicates. Statistically significant differences in the parameters measured within each species were determined with two‐way ANOVA with a Tukey's post hoc test (*p* < 0.05) and are represented as different letters.

All three species can exist as single motile cells or multi‐cell palmelloids (Figure 1), and we observed that light availability (but not salinity) had a significant effect on algal morphology (Table S2). While cell size (~6.9 μm) did not change significantly in the lacustrine *C. priscui* cultures in response to experimental conditions (Figure 2b), the palmelloid content was ~4× higher in cultures acclimated to high light compared to those at lower light intensities (Figure 2c). The marine *C. malina* had significantly larger single cells (~5.6 μm) and ~4× more palmelloid colonies when cultured with higher light availability, in comparison with cultures grown at lower light (~5.0 μm) (Figure 2b,c). Finally, the snow alga *C. klinobasis* had the largest cells, with a significant cell size difference and 5× higher palmelloid content between high light (~11.7 μm) and low light (~10.9 μm) acclimated cultures (Figure 2b,c).

Light availability was a major environmental contributor of steady‐state pigment content in all three species, but we demonstrated that salinity has a species‐specific effect (Tables 2 and S2). All three species accumulated ~1.5–2× more Chl cell^−1^ when acclimated to low light intensity compared to cultures grown with higher light, regardless of salinity (Table 2). While *C. klinobasis* typically accumulated 2–4× more chlorophyll compared to *C. malina* and *C. priscui*, this is likely a reflection of the larger cell size in this species (Figure 2b). Acclimation to limited light led to lower Chl *a/b* ratios in all species, but salinity had a significant effect only in *C. malina* cultures. This Arctic alga grown at higher salinity exhibited small but significant (~7%) increase in Chl *a/b* ratios compared to cultures grown at lower salinity, when acclimated to equivalent light levels (Tables 2 and S2). Car/Chl ratios were also regulated by light in all three species, where algae acclimated to higher light exhibited a higher ratio of the two pigments (Table 2). While salinity had no effect on the pigment composition balance in *C. malina* , Car/Chl ratios were affected by both variables in *C. priscui* and *C. klinobasis*. In both cases, cultures acclimated to high salinity accumulated more carotenoids compared to those acclimated to low salinity at the equivalent light, leading to a significant decrease (5%–13%) in the Car/Chl ratios (Table 2).

**TABLE 2:** The effects of salinity and light on the pigment content of *C. priscui*, *C. malina* and *C. klinobasis*.

	HS‐LL	HS‐HL	LS‐LL	LS‐HL
*C. priscui*				
Chl/cell (pg)	3.10 ± 0.58^a^	2.43 ± 0.57^a^	3.66 ± 0.96^a^	2.30 ± 0.2^a^
Chl *a/b*	2.03 ± 0.09^b^	3.08 ± 0.15^a^	2.18 ± 0.07^b^	3.28 ± 0.21^a^
Car/Chl	0.41 ± 0.01^b^	0.44 ± 0.01^a^	0.39 ± 0.01^c^	0.42 ± 0.01^ab^
*C. malina*				
Chl/cell (pg)	2.21 ± 0.25^a^	1.15 ± 0.12^b^	2.17 ± 0.31^a^	1.07 ± 0.05^b^
Chl *a/b*	1.92 ± 0.03^d^	3.02 ± 0.07^b^	2.06 ± 0.02^c^	3.26 ± 0.07^a^
Car/Chl	0.45 ± 0.01^b^	0.51 ± 0.01^a^	0.44 ± 0.01^b^	0.5 ± 0.01^a^
*C. klinobasis*				
Chl/cell (pg)	5.01 ± 0.29^ab^	3.62 ± 0.57^b^	9.51 ± 3.79^a^	4.44 ± 0.83^a^
Chl *a/b*	2.67 ± 0.08^b^	4.18 ± 0.05^a^	2.61 ± 0.05^b^	3.92 ± 0.37^a^
Car/Chl	0.46 ± 0.01^c^	0.6 ± 0.02^a^	0.42 ± 0.01^d^	0.52 ± 0.01^b^

*Note:* In all cases, algae were cultured at 4°C and acclimated to high salinity (HS), low salinity (LS), high light (HL), or low light (LL). Data are means ± SD of at least three biological replicates each with at least four technical replicates. Statistically significant differences within each species were determined with two‐way ANOVA with a Tukey's post hoc test (*p* < 0.05) and are represented as different letters.

### 3.3 Rapid Growth Correlates With Increased Sensitivity to Temperature Stress

Culturing at different salinity and light levels affects growth, morphology, and pigment content in *polar Chlamydomonas* (Figure 2, Table 2) but how acclimation to these conditions affects algal responses to temperature stress has not been examined before. To test this, we exposed algae acclimated to four different conditions (Table 1) to temperature stress. To compare the temperature sensitivity of the three species, we used an equivalent stress model instead of an equal temperature model. *C. priscui* is the only psychrophilic alga that has been examined under temperature stress (24°C) (Possmayer et al. 2011; Cvetkovska, Zhang, et al. 2022). Thus, we exposed *C. malina* and *C. klinobasis* to a range of non‐permissive temperatures and selected temperatures at which the kinetics of chlorophyll bleaching and cell death most closely mirrored those previously established in *C. priscui*. We showed that both *C. malina* (Figure S3c) and *C. klinobasis* (Figure S3d) cultures incubated at 22°C lose viability at rates similar to those of *C. priscui* at 24°C when acclimated to equivalent light and salinity levels. This suggests that these two species, despite originating from dynamic environments, are more sensitive to temperature stress compared to *C. priscui*.

We demonstrated that acclimation to different environmental conditions has a significant effect on the resilience of psychrophilic algae to long‐term temperature stress (Figure 3). In all three species, temperature‐induced cell death (Figure 3a–c) and chlorophyll loss (Figure 3d–f) occurred most rapidly in cultures acclimated to low salinity and high light intensity (3–4 days to 100% viability loss; 4–5 days to 100% chlorophyll loss), conditions that also led to the fastest growth rates prior to exposure to elevated temperature (Figure 2a). Conversely, slow‐growing cultures acclimated to high salinity and low light intensity (Figure 2a) exhibited robust resilience to temperature stress and gradually lost viability (Figure 3a–c) and chlorophyll (Figure 3d–f) over ≥ 15 days.

**FIGURE 3:**
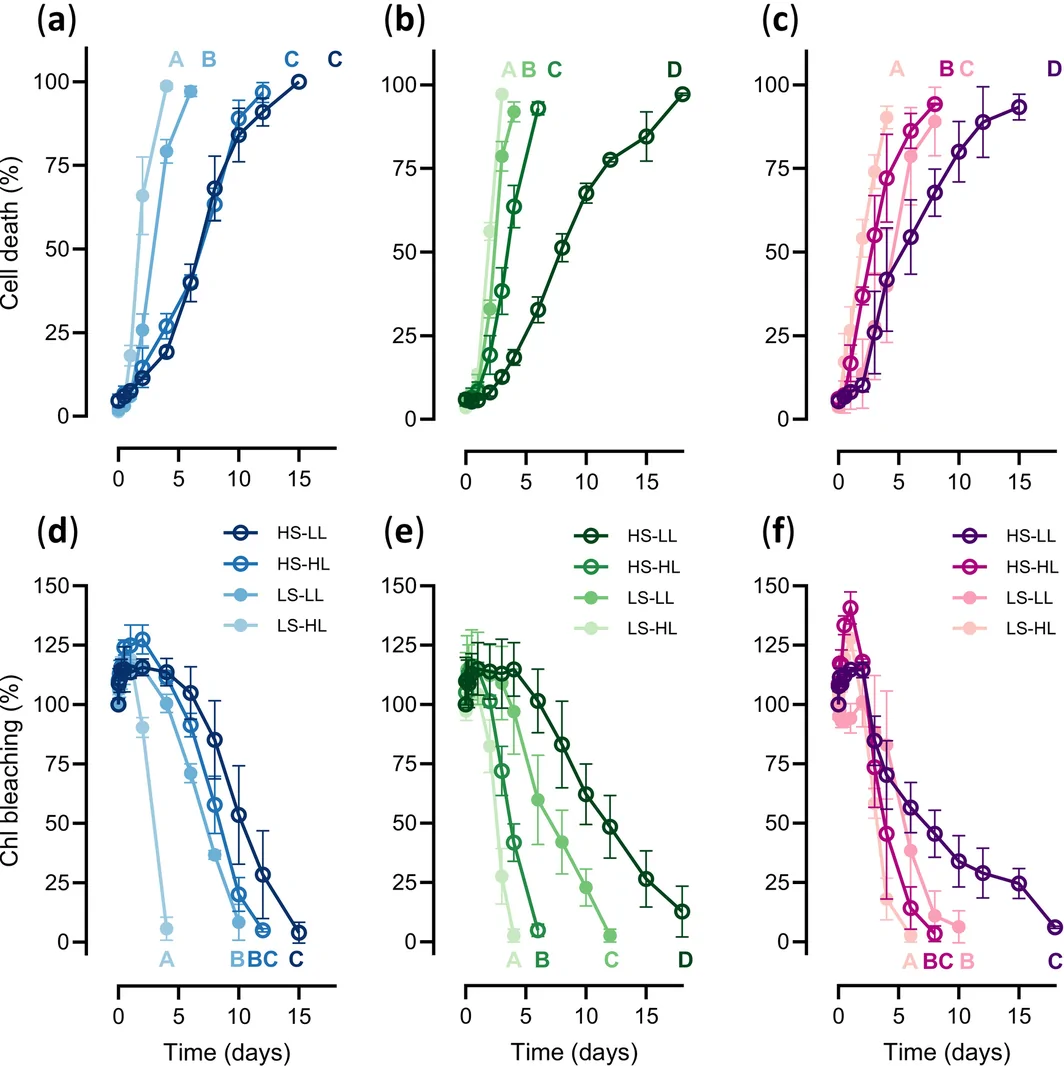
The kinetics of cell death (a, b, c) and chlorophyll bleaching (d, e, f) in *C. priscui* (a, d; blue), *C. malina* (b, e; green) and *C. klinobasis* (c, f; mauve) exposed to non‐permissive temperature. In all cases, algae were cultured at 4°C and acclimated to high salinity (HS), low salinity (LS), high light (HL) or low light (LL). Temperature stress was initiated at the mid‐exponential growth phase (OD_750_ = 0.3–0.4) and corresponds to *t* = 0 days. Cell death was calculated as a percent of algal cells stained with the live‐dead stain SYTOX Green. Chlorophyll bleaching was calculated as the proportion of chlorophyll present at each time point compared to levels prior to exposure to temperature stress (0 days). Data are means ± SD of at least three biological replicates, each with four technical replicates. Statistically significant differences in the parameters measured within each species were determined by a linear model with a Tukey's post hoc test (*p* < 0.05) and are represented as different letters.

In addition to these robust trends, the interaction between light and salinity had a more subtle, species‐specific effect. Salinity was the main driver behind temperature resilience in the lacustrine *C. priscui*, where cultures acclimated to 700 mM NaCl exhibited slow cell death and chlorophyll loss kinetics, regardless of the light intensity (Figure 3a,d, Table S3). Light intensity had a significant effect on the kinetics of chlorophyll loss in this alga, but only in cultures acclimated to low salinity (Figure 3d, Table S3). Both salinity and light intensity significantly affected the viability of the marine *C. malina* during temperature stress. Cultures acclimated to low salinity prior to temperature stress suffered more rapid heat‐induced cell death compared to those acclimated to higher salinity. Conversely, higher light‐acclimated cultures lost chlorophyll faster during heat stress, compared to those acclimated to low light (Figure 3e, Table S3). Finally, light intensity appeared to be the main factor behind temperature sensitivity in the snow alga *C. klinobasis*. Algae acclimated to higher light exhibited more rapid rates of cell death and chlorophyll loss compared to those grown at lower light, regardless of the salinity (Figure 3c,f, Table S3).

### 3.4 Acclimation to Light and Salinity Affects PSII Photochemistry During Temperature Stress

Photosynthesis is highly sensitive to environmental stress and is affected before chlorophyll loss or cell death becomes apparent (Nievola et al. 2017; Lodeyro et al. 2021). All three psychrophilic species had robust photosynthetic capacity in all conditions when grown at 4°C. In all cases, F_V_/F_M_ (0.7–0.8; Figure 4a–c) and ETR (11–14 μmol m^−2^ s^−1^; Figure 4d–f) values were within the normal range for green algae (Malapascua et al. 2014). The exception was *C. priscui* acclimated to high salinity, with lower *F*_*V*_/*F*_*M*_ (0.52–0.56; Figure 4a) and ETR (8–9 μmol m^−2^ s^−1^; Figure 4d). We also observed lower F_V_/F_M_ values (0.64–0.67; Figure 4c) in *C. klinobasis* cultures acclimated to higher light intensity, although this alga maintained high ETR rates under these conditions (11.2–13.3 μmol m^−2^ s^−1^; Figure 4f).

**FIGURE 4:**
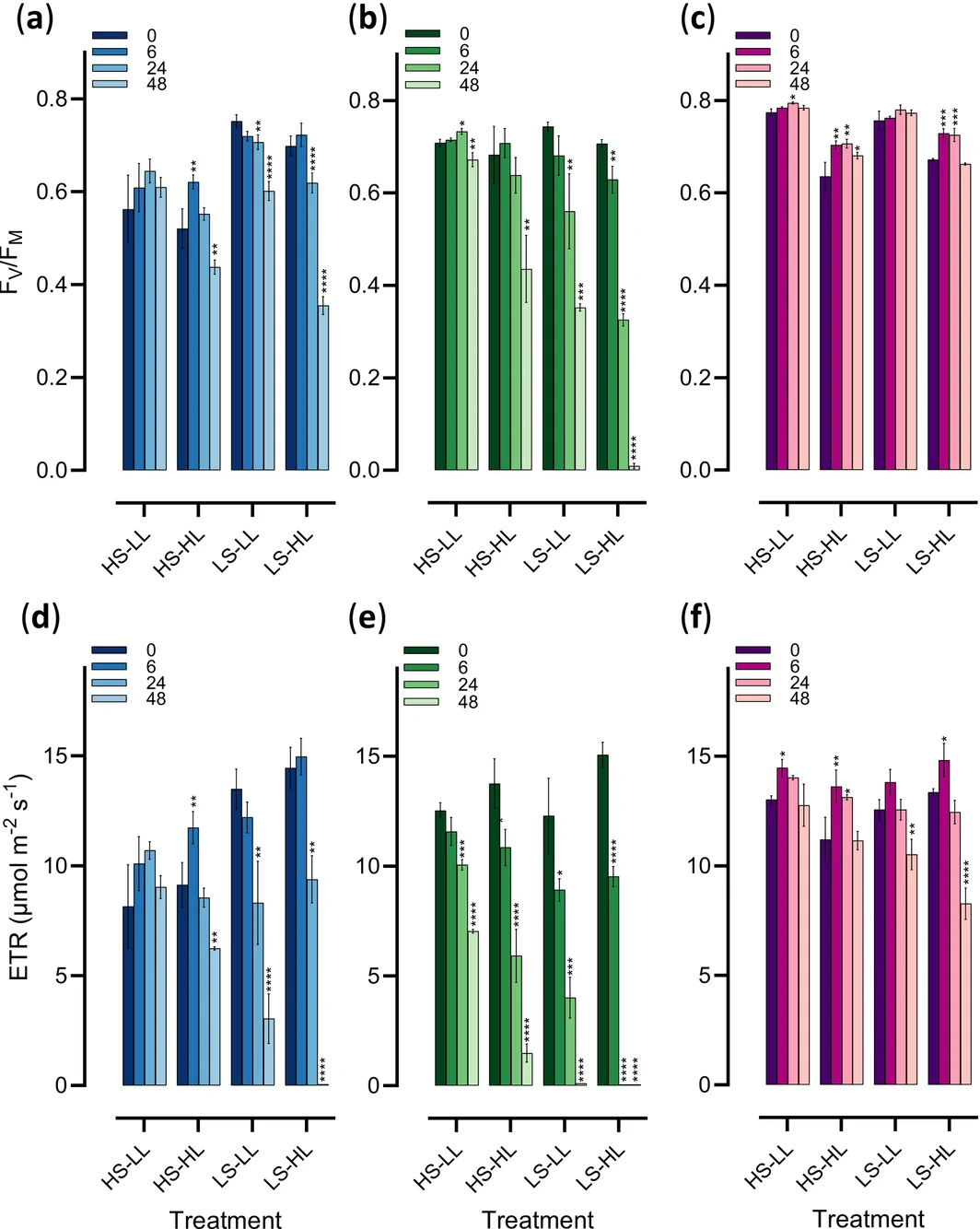
The maximum PSII photochemical efficiency (F_V_/F_M_; a, b, c) and relative electron transport rate (ETR; d, e, f) in *C. priscui* (a, d; blue), *C. malina* (b, e; green) and *C. klinobasis* (c, f; mauve) exposed to non‐permissive temperature. In all cases, algae were cultured at 4°C and acclimated to high salinity (HS), low salinity (LS), high light (HL) or low light (LL). Temperature stress was initiated at the mid‐exponential growth phase (OD_750_ = 0.3–0.4) and corresponds to *t* = 0 h. Data are means ± SD of at least three biological replicates, with at least four technical replicates each. Statistical significance compared with the value prior to stress application (*t* = 0) within each species were determined with three‐way RM ANOVA with a Dunnett's post hoc test (* = < 0.05, ** = < 0.01, *** = < 0.001, **** = < 0.0001). Detailed statistical analyzes can be found in Table S3.

Salinity and light intensity significantly affected photochemistry during temperature stress in all three species (Table S3), but the magnitude of this effect depended on the initial acclimation conditions. Mirroring chlorophyll loss and cell death trends, photosynthetic parameters measured in slow‐growing algal cultures acclimated to high salinity and low light were the least affected by temperature stress. *C. priscui* (Figure 4a,d) and C. *klinobasis* (Figure 4c,f) cultures did not exhibit s decrease in F_V_/F_M_ and ETR over 48 h at increased temperature. Instead, we observed a small increase in these parameters during the first 24 h, although these values were significant only in *C. klinobasis* (35% increase in F_V_/F_M_ at 24 h, Figure 4c; 10% increase in ETR at 6 h, Figure 4f). *C. malina* was the most sensitive to temperature increase at the level of the photosynthetic apparatus, with significant decreases in both parameters (5% decrease in *F*_*V*_/*F*_*M*_, Figure 4b; 44% decrease in ETR, Figure 4e) after 48 h of temperature stress.

Fast‐growing cultures acclimated to low salinity and higher light were the most sensitive to temperature stress. Both *C. priscui* (Figure 4a) and *C. malina* (Figure 4b) experienced a significant decrease in maximum quantum efficiency after 48 h (49% and 99% decrease in Δ*F*_*V*_/*F*_*M*_, respectively). Rates of electron transport were severely affected and not detectable after 48 h in *C. priscui* (Figure 4b) and only 24 h in *C. malina* (Figure 4e). These species acclimated to a combination of high salinity and high light, or low salinity and low light, exhibited similar decreases in *F*_*V*_/*F*_*M*_ and ETR, albeit to a smaller extent (Figure 4a,b,d,e). *C. klinobasis* was the least affected by temperature stress. While ETR rates were moderately decreased by 48 h of temperature stress in cultures acclimated to low salinity and higher light (32%, Figure 4f), *F*_*V*_/*F*_*M*_ exhibited a transient increase during this period (7%; Figure 4c).

Light energy absorbed by PSII can be used for photochemistry [Y(II)] or dissipated, either through regulated processes and antenna quenching [Y(NPQ)] or through non‐regulated processes [Y(NO)] (Kramer et al. 2004). Our results indicate that energy partitioning is sensitive to temperature increases in polar *Chlamydomonas* and highly dependent on the salinity and light acclimation prior to stress (Figure 5, Table S4). Incubation at non‐permissive temperature caused a significant increase in Y(NPQ) and Y(NO) at the expense of Y(II) in both *C. priscui* and *C. malina* at 48 h, a response that was dependent on the acclimation to both light and salinity (Table S3). The exception to this trend occurred in *C. priscui* acclimated to high salinity and low light, where Y(II) increased after 48 h of stress (10%; Figure 5a, Table S4). Acclimation to all other growth conditions led to decreases in Y(II), although the effect was moderate (32% decrease) in cultures grown at high salinity and high light, and severe in those acclimated to low salinity (77%–100% decrease; Figure 5a, Table S4). The Arctic *C. malina* suffered significant stress‐induced decrease in Y(II) (Figure 5b, Table S4). While this decrease was moderate in algae acclimated to high salinity and low light (44%), we observed a precipitous decline in all other conditions after 48 h (~89%–100%). The decline was particularly severe in algae grown at low salinity and high light intensity, with a complete loss of photochemical quenching by 24 h of temperature stress (Figure 5b, Table S4).

**FIGURE 5:**
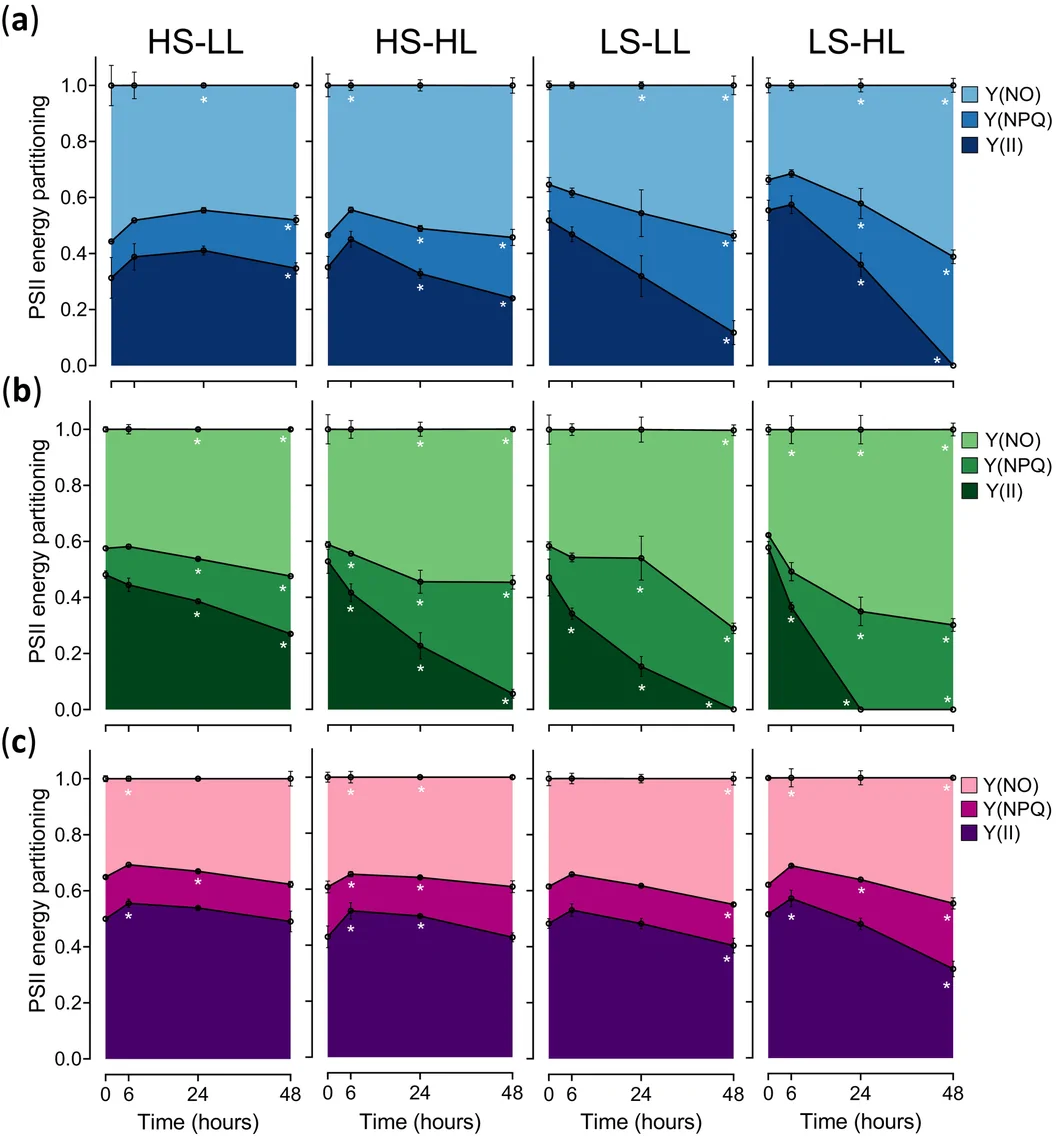
Energy partitioning at PSII in *C. priscui* (a; blue), *C. malina* (b; green), and *C. klinobasis* (**c**; mauve) exposed to non‐permissive temperature. The total absorbed light is distributed between photochemical yield of PSII Y(II), non‐photochemical quenching Y(NPQ), and non‐regulated dissipation Y(NO). In all cases, algae were cultured at 4°C and acclimated to high salinity (HS), low salinity (LS), high light (HL) or low light (LL). Temperature stress was initiated at the mid‐exponential growth phase (OD_750_ = 0.3–0.4) and corresponds to *t* = 0 h. Data are means ± SD of at least three biological replicates, with at least four technical replicates each. Statistically significance compared with the value prior to stress application (*t* = 0) within each species were determined with three‐way RM ANOVA with a Dunnett's post hoc test (* = < 0.05). Detailed statistical analyzes are available in Table S4.

Finally, photochemistry in the snow alga *C. klinobasis* was the least affected by temperature stress, compared to its halotolerant relatives. Incubation at non‐permissive temperature caused a rapid but transient increase in Y(II) by 6 h (10%–18%) accompanied by a decrease in Y(NO) (12%–13%) in all culturing conditions (Figure 5c, Table S4). Photochemical quenching was negatively affected by 48 h only in the cultures acclimated to low salinity (16% and 38% at low and high light respectively; Figure 5c, Table S4).

## 4 Discussion

### 4.1 Close Relatives From Opposite Sides of the Globe

Our phylogenetic analysis places all three *Chlamydomonas* species examined here in the Moewusinia and Monadinia clades within the Chlamydomonadales, an order that represents one of the most taxonomically and phylogenetically complex groups of green algae (Nakada et al. 2008; Demchenko et al. 2012). The taxonomic identity of the model Antarctic psychrophile *C. priscui* has experienced much change (Morgan‐Kiss and Guiry 2024) but thorough phylogenetic analysis placed this alga in a unique lineage within the Moewusinia clade (Possmayer et al. 2016). A close phylogenetic relationship with the marine *Chlamydomonas parkeae* (SAG 24.89) suggested a mesophilic ancestor prior to the arrival of *C. priscui* in Lake Bonney (Possmayer et al. 2016). Here, we demonstrate that the Arctic marine *C. malina* (RCC2488, Balzano et al. 2012; Morales‐Sánchez et al. 2020a) and the Antarctic marine C. sp. WAP05/30 (Soto et al. 2023) are the closest known relatives of *C. priscui* (Figures 1, S1 and S2), raising other possibilities for the ancestry of this group. The Arctic snow alga *C. klinobasis* also shares a close relationship with both Arctic and Antarctic psychrophiles within the Monadinia clade (Figures 1, S1 and S2), including *Chlamydomonas* sp. ARC (Eddie et al. 2008), *M. antarctica* (Trentin et al. 2022), *C*. sp. ICE‐L, ICE‐W (Liu et al. 2006), ICE‐MDV (Cook et al. 2019) and KNF0022 (Jung et al. 2016). These patterns suggest the possibility of “Polar clades” within the Chlamydomonadales enriched in psychrophilic species.

These close phylogenetic relationships, despite geographical distance, raise several possibilities that could explain the ancestry of psychrophilic green algae. For example, the Arctic‐Antarctic split within this group could result from ancient climate history rather than modern geographical connectivity. Snow‐ and ice‐associated *Chlamydomonas* are often capable of forming life stages resilient to desiccation, freezing, and high UV exposure (Hoham and Remias 2020) that could facilitate long‐distance atmospheric or oceanic dispersal, particularly during colder geological periods (e.g., the Pleistocene glaciation, Clark et al. 2009). The extreme conditions and prolonged isolation between the Arctic and Antarctic could then favor a narrow set of pre‐adapted lineages and result in a closely related but geographically isolated species that occupy comparable polar niches (Dorrell et al. 2023). Similar theories have been suggested for the dispersal of cold‐adapted Trebouxiophyceae, diatoms and foraminifers (Darling et al. 2000; Vyverman et al. 2007; Hodač et al. 2016; Segawa et al. 2018, 2023), and such events could have shaped modern polar *Chlamydomonas* diversity as well. This genusmay be especially predisposed to bipolar evolutionary patterns: species within this group occupy a wide range of aquatic habitats, including snow, glacier ice, fresh waters, brackish and marine environments, and have repeatedly developed traits for cold tolerance, osmotic flexibility, and stress resilience (Leliaert et al. 2012; Hoham and Remias 2020). Molecular clock analyzes are currently limited by sparse taxon sampling, leaving a high likelihood of unsampled diversity (Rappaport and Oliverio 2023), and broader genomic representation will enable divergence‐time estimates across this group in the future.

### 4.2 Less Extreme Conditions Promote Algal Growth and Phenotypic Change

Light and salinity are major environmental drivers for microbial growth in polar environments (Li et al. 2019; Duncan, Nielsen, et al. 2024; Duncan, Søreide, et al. 2024; Ahmed et al. 2025). All three psychrophiles examined here exhibited the slowest growth rates when acclimated to shading (Figure 2a), conditions most often associated with ice‐ or snow‐covered environments (Stibal et al. 2007; Smoot and Hopcroft 2017; Patriarche et al. 2021). Conversely, their population increased the fastest when acclimated to less extreme conditions. Light was a major driver of high growth rates, particularly when combined with lower salinity (Figure 2a). These results are consistent with published lab‐based studies on axenic algal cultures (Takizawa et al. 2009; Pocock et al. 2011; Lee et al. 2018; Suzuki et al. 2019; Morales‐Sánchez et al. 2020a; Stahl‐Rommel et al. 2021), but they are also reminiscent of recent ecological observations in polar environments. The loss of the ice‐ and snow‐cover, the freshening of marine habitats due to warming, and the consequent increase in greening and primary productivity have been well documented in both Arctic (Arrigo et al. 2012; Myers‐Smith et al. 2020; Castellani et al. 2022; Manizza et al. 2023; Wolf et al. 2024) and Antarctic habitats (van Leeuwe et al. 2018; Del Castillo et al. 2019; Gray et al. 2020; Roland et al. 2024).

In addition to high growth rates, we observed higher palmelloid content in all three psychrophiles acclimated to higher light levels, regardless of salinity (Figure 2c), an effect that may lead to an underestimation of the true extent of cell proliferation. Palmelloid formation in *Chlamydomonas* is thought to arise from incomplete release of the daughter cells following asexual division, resulting in a single colony of multiple cells contained within an extracellular matrix (Ng et al. 2022; Suwannachuen et al. 2023). Thus, increased palmelloid formation under HL may mask an even greater stimulation of cell proliferation than suggested by the measurements of OD or cell counts, as these methods cannot distinguish between the individual cells encapsulated within the colony. Palmelloid formation may also be a general stress response in green algae and has been observed upon exposure to pollutants, salinity stress, nutrient deficiency, and grazers (Lurling and Beekman 2006; Ratcliff et al. 2013; Khona et al. 2016; Cheloni and Slaveykova 2021; Pascual et al. 2025). In addition, recent evidence suggests that this morphological structure may be photoprotective. The mesophilic *C. reinhardtii* utilized the formation of palmelloids and aggregates to shield internal cells from excessive light and promote increases in antioxidant content (Suwannachuen et al. 2023). The Antarctic *C. priscui* grown at supraptimal temperatures (16°C) induced the formation of palmelloids with modified organization of the photosynthetic apparatus and enhanced capacity for quenching of excess absorbed light (Szyszka‐Mroz et al. 2022; Hüner et al. 2023). Similar morphology has been reported in alpine algal biofilms, suggesting a protective role against excessive light and UV exposure in snow communities (Karsten and Holzinger 2014). We observed a light‐driven increase in palmelloid content in all three species, regardless of their native habitat (Figure 2c), suggesting that a change in phenotype from a motile single cell to a non‐motile colony may be a general photoprotective response in psychrophilic green algae. However, it should be noted that the light intensities used in our work are unlikely to induce severe photodamage. For instance, *C. priscui* exhibited robust growth and enhanced phototolerance at 250 μmol m^−2^ s^−1^, light intensities equivalent to those measured on the surface of open moats in Antarctic lakes (Popson et al. 2024), and algae growing at the snow‐air interface routinely experience very high irradiances (1000–2000 μmol m^−2^ s^−1^) (Hoham and Remias 2020). Thus, a comprehensive assessment of palmelloid formation in psychrophilic algae requires testing at light intensities near the upper permissible range, where the photoprotective role of this phenotype and its effect on algal population growth are most likely to become apparent.

### 4.3 Resilience to Temperature Stress Is Dependent on Acclimation to Light and Salinity

A highlight of this study is the observation that all three psychrophiles acclimated to less extreme conditions (higher light intensity, lower salinity) exhibit rapid growth, but also the highest sensitivity to temperature stress. All three species acclimated to these “relaxed” conditions lost viability and chlorophyll within 3–4 days when challenged with non‐permissive temperatures (Figure 3). High growth rates also correlate with a significant and rapid deterioration of *F*_*V*_/*F*_*M*_ (Figure 4a–c), electron transport rates (Figure 4d–f), and photochemical yields (Figure 5a–c) within 24–48 h of temperature stress. In contrast, slow‐growing algal cultures acclimated to high salinity and low light intensity were much more robust. Complete loss of viability and chlorophyll required ~15 days of temperature stress (Figure 3), and these cultures maintained the efficiency of their photosynthetic apparatus (Figures 4 and 5) over 48 h at increased temperatures. This pattern suggests that enhanced temperature resilience is associated with the physiological state established during HS‐LL acclimation, rather than slow growth per se. Indeed, PSII heat tolerance is increasingly recognized as a dynamic trait that can be strongly influenced by prior acclimation conditions in land plants (Posch et al. 2026), and similar insights are emerging in green algae (Barati et al. 2019; Zhang et al. 2022). It has been postulated that the mechanistic basis of PSII acclimation may lie in the stabilization of the oxygen evolving complex by stress‐induced molecular chaperones (Schroda et al. 1999; Hu et al. 2015; Chen et al. 2017), accumulation of sugar solutes that may alter the melting point of the thylakoid membranes (Andersen et al. 2011; Zhu et al. 2024), or increased contribution of cyclic electron flow (CEF) around PSI that may alleviate PSII over‐reduction during periods of environmental stress (Yamori and Shikanai 2016). While these priming mechanisms are very likely species‐specific, the patterns of *F*_*V*_/*F*_*M*_ and ETR decline (Figure 4) suggest that both salinity and light contribute to this acclimation state at the level of the photosynthetic apparatus in psychrophilic green algae. Their relative importance, however, differs among the examined species.

*C. priscui* is the only psychrophilic alga that has been examined in the context of temperature stress, but all previous studies were done on cultures acclimated to low salinity and higher light. This alga constitutively accumulates stress‐related metabolites and proteins (e.g., heat shock proteins, antioxidants) during low temperature growth, likely as an adaptation to the extreme conditions in Lake Bonney, but fails to further increase their levels during short‐term acute temperature stress (Cvetkovska, Zhang, et al. 2022). Longer exposure to heat leads to loss of Y(II), consequent increases in Y(NO) and Y(NPQ) and ultimately rapid cell death (Possmayer et al. 2011). These studies suggested that adaptations that confer a benefit for life at the extreme may also limit the ability of psychrophilic algae to respond to environmental disturbances (Cvetkovska, Vakulenko, et al. 2022), but here we demonstrate that these responses are highly dependent on algal acclimation to different light and salinity levels. Our work paves the way towards a detailed examination of the molecular basis that underlies the sensitivity of polar algae to environmental change and the roles of salinity and light in stress resilience.

While these general trends hold true in all three psychrophiles examined here, we also show that the interaction between light, salinity, and temperature is complex. For instance, our data suggest that salinity is the main driver of temperature sensitivity in *C. priscui*, while light intensity more strongly affects stress resilience in *C. klinobasis*. The temperature sensitivity in *C. malina* appears to be governed by both light and salinity (Figure 4, Table S3). These patterns could reflect the species' native habitats. *C. priscui* is endemic to the perennially ice‐covered deep waters of Lake Bonney. This is an extremely light‐limited environment with dramatic increases in conductivity within a sharp chemocline at ~17–20 m, which coincides with the depth at which *C. priscui* was isolated (Neale and Priscu 1995; Patriarche et al. 2021). The freshwater alga *C. klinobasis* was isolated from ephemeral snowfields on Svalbard Island (Hulatt et al. 2017), a dynamic oligotrophic environment that experiences significant light variability: extremely high irradiation on the surface with rapid light attenuation within the deeper snow layers (Stibal et al. 2007). Finally, the Arctic marine alga *C. malina* is native to the Beaufort Sea (Balzano et al. 2012), a highly dynamic habitat characterized by a thick ice cover in the winter, and thinner ice packs and open waters during the summer. In addition, this environment receives significant freshwater and nutrient inputs from the surrounding land masses (Tremblay et al. 2008; Rheinlænder et al. 2024). Thus, the responses of these psychrophiles to temperature stress are likely shaped by life at cold, but otherwise very different, native habitats.

Cross‐tolerance between salinity and heat has been reported in mesophiles, including a similar response in the green algae *Dunaliella* sp. and *Tetraselmis striata*, which were progressively more resilient to heat stress when cultured at increasing salinities (Henley et al. 2002; Santos et al. 2025). Similarly, exposure to moderate salinities leads to improved resilience in plants (Rivero et al. 2014; Pushpavalli et al. 2020; Bryant et al. 2024) and coral endosymbionts (Gegner et al. 2017), suggesting a conserved response. The mechanisms behind this beneficial effect are not fully understood and are likely species‐specific, but moderate salinity stress enhances the accumulation of protective metabolites (e.g., proline, glycine, sucrose), antioxidants (e.g., ascorbate), enhanced thermal adaptation via NPQ, and cytosolic Ca^2+^‐related signaling cascades (Rivero et al. 2014; Shaar‐Moshe et al. 2017; Ji et al. 2018; Guo et al. 2022; Vineeth et al. 2023; Bryant et al. 2024) likely priming for broader stress resilience. Specifically in green algae, salinity‐based heat stress priming has been associated with increased photosynthetic capacity and glycerol biosynthesis (Liska et al. 2004), and enhanced accumulation of heat shock proteins (HSPs) that may stabilize the photosynthetic machinery (Shetty et al. 2019; Santos et al. 2025). High salinity‐acclimated *C. priscui* cultures accumulate high levels of antioxidants and proline, and exhibit enhanced CEF around PSI (Kalra et al. 2020, 2023; Stahl‐Rommel et al. 2021). This alga also exhibits constitutive HSP accumulation (Cvetkovska, Zhang, et al. 2022), which likely contributes to enhanced heat stress resilience in conjunction with other salinity‐enhanced protective pathways. Similar mechanisms likely confer a broad‐spectrum salinity‐derived resilience to temperature stress in all psychrophiles examined here (Figures 4 and 5).

The interaction between light and elevated temperature is considered antagonistic in photosynthetic organisms, as higher irradiances can exacerbate heat stress by increasing PSII excitation pressure, accelerating photoinhibition, and increasing chloroplast‐derived ROS accumulation (Allakhverdiev et al. 2008; Gollan and Aro 2020; Zahra et al. 2023). Possmayer et al. (2011) demonstrated that a shift to a non‐permissive temperature under growth irradiance (100 μmol m^−2^ s^−1^) led to significantly increased kinetics of cell death in *C. priscui*, compared to equivalent temperature stress applied to cultures in the dark. While this alga is capable of long‐term acclimation to higher irradiance (100–250 μmol m^−2^ s^−1^) due to its robust antioxidant system, high NPQ capacity, and the protective role of CEF (Szyszka et al. 2007; Popson et al. 2024; Poirier et al. 2025), these mechanisms are likely insufficient to counteract the multifactorial effect of combined higher irradiance and temperature stress (Figures 4a–d and 5a). Conversely, acclimation to lower light may reduce excitation pressure when photosynthetic metabolism is subsequently disrupted by high temperature, contributing to the greater ability to maintain photosynthetic function observed in slow‐growing cultures.

While similar processes are likely to operate in *C. malina* (Figures 4b–e and 5b), the effects of light may differ in *C. klinobasis* (Figures 4c–f and 5c). Among the psychrophiles examined here, this snow alga displayed the highest temperature stress resilience at the level of photosynthesis and maintained photosynthetic efficiency over 48 h in all conditions (Figure 5). Snow algae have well‐characterized protection against extreme irradiation due to a robust capacity for the synthesis of astaxanthin and other carotenoids. These pigments with photoprotective and antioxidant functions may physically shade organelles from light damage and act as metabolic sinks (Hoham and Remias 2020). Such mechanisms may contribute to the stress resilience of *C. klinobasis* observed here.

## 5 Conclusion and Implications for Polar Photosynthetic Life During Climate Change

Our work is the first to directly correlate the acclimation to different light and salinity levels with temperature stress resilience in psychrophiles. The effects of temperature, light, and salinity on the physiology of psychrophilic algae have been examined in a handful of studies (Morales‐Sánchez et al. 2020a, 2020b; Young and Schmidt 2020; Cvetkovska, Vakulenko, et al. 2022; Hüner et al. 2023; Morgan‐Kiss et al. 2024), but previous work has largely centered on a single environmental variable. Light and salinity are the main drivers of algal diversity and productivity in polar environments, and the ones that rapidly change with the loss of ice cover in the warming polar regions (Castendyk et al. 2016; Lehnherr et al. 2018; Li et al. 2019; Obryk et al. 2019; Meiners et al. 2025). Cold‐water organisms will be further challenged by short‐term but severe episodes of anomalous weather patterns, including more frequent extreme heat waves (Huang et al. 2021) with recorded temperatures rising ~40°C above the yearly average (Constable 2022; Blanchard‐Wrigglesworth et al. 2023).

Our results suggest that fast‐growing psychrophilic algae acclimated to lower salinities and increasing light intensities are at particular risk (Figure 6). This is likely to have major implications in polar habitats, and particularly in ice‐covered Antarctic lakes and snow fields dominated by psychrophilic *Chlamydomonas* (Li et al. 2019; Hoham and Remias 2020; Morgan‐Kiss et al. 2024). Indeed, the once highly abundant *C. priscui* (Neale and Priscu 1995) is only rarely detected in the Antarctic Lake Bonney in recent surveys (Li et al. 2019; Morgan‐Kiss et al. 2024) and may be one of the casualties of climate change. Our work was done on axenic cultures, but natural microbial communities are complex, and many species are psychrotolerant rather than psychrophilic (Hüner et al. 2022). Rising temperatures may give a competitive advantage to psychrotolerant algae that survive in the cold but have growth maxima at temperatures ≥ 20°C; thus, fine‐grained analyzes on the physiology of psychrophilic algae faced with changing conditions must be complemented with long‐term observations of algal population dynamics in the polar regions.

**FIGURE 6:**
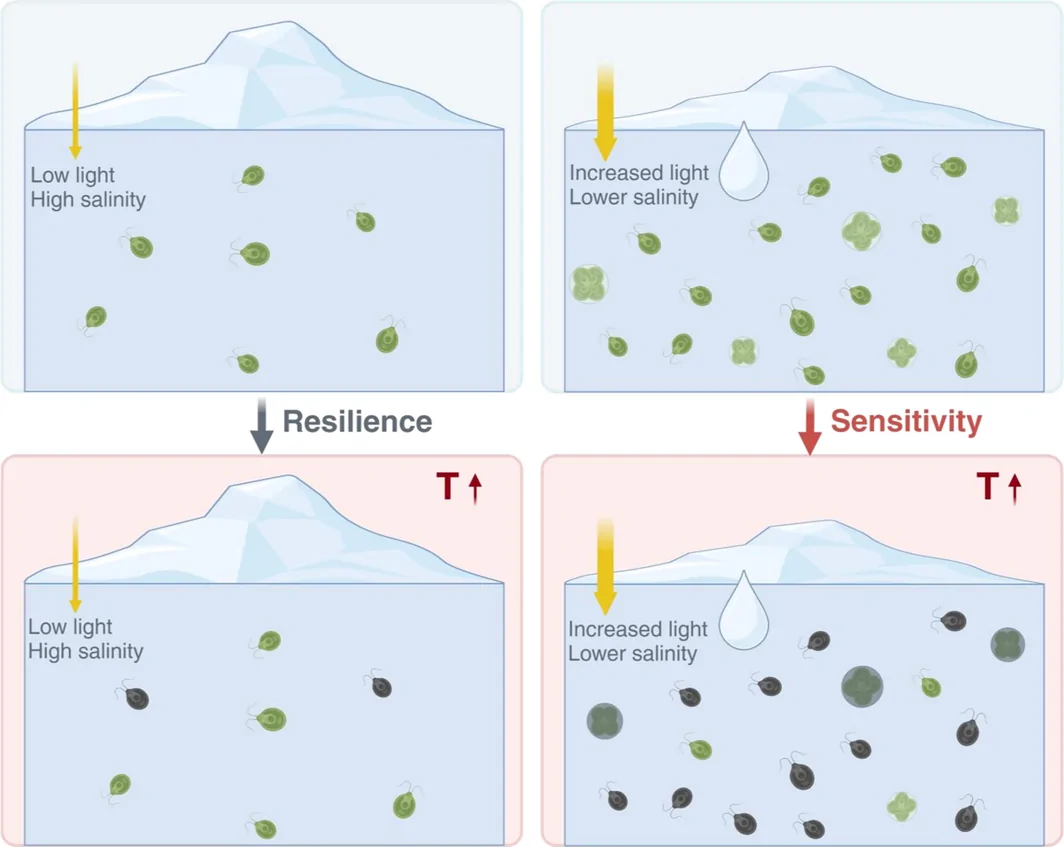
A model representing the responses of psychrophilic *Chlamydomonas* to a changing environment. Green algae acclimated to higher salinity and lower light intensities exhibit slow growth (top left) but maintain high resilience to subsequent lethal heat stress (bottom left). Conversely, green algae acclimated to increased light intensities and decreased salinities, conditions that may result from ice and snow melt, grow rapidly (top right) but are sensitive to lethal heat stress and display rapid cellular death (bottom right).

Natural habitats are characterized by co‐occurring or sequential stressors, and a conceptual framework known as the multifactorial stress combination (MFSC) principle has been proposed to address the effect of multiple conditions on an organism (Zandalinas and Mittler 2022). MFSC responses have been explored in plants (Zandalinas et al. 2021) and soil microbial communities (Rillig et al. 2019), but how multifactorial and dynamic conditions affect psychrophilic algae is still elusive. Our work provides the first advancement in mapping the responses of polar algae to a changing environment and opens the doors to future studies on the acclimation and stress resilience in these important primary producers.

## Author Contributions

P.O. and M.C. designed the study and interpreted the data. P.O. performed all physiology experiments and analyzed the data. N.B. assisted with algal culturing and performed the initial growth characterization of *C. malina* and *C. klinobasis*. A.S. and L.K. contributed to sequence data collection and phylogenetic analyzes. P.O. and M.C. wrote the original draft. All authors edited and approved the manuscript.

## Funding

This work was supported by the Natural Sciences and Engineering Research Council of Canada (RGPIN‐2019‐05763), Canada Foundation for Innovation (39233), University of Ottawa, The Government of Ontario, the Ontario Graduate Scholarship.

## Disclosure

No generative AI tools were used in data generation, analysis, interpretation of the results, or writing of the manuscript.

## Conflicts of Interest

The authors declare no Conflicts of Interest.

## Supporting information


**Figure S1:** Phylogenetic tree of 63 *RBCL* nucleotide sequences inferred from using maximum likelihood analysis is shown to scale (substitutions per site), with branch values representing bootstrap support. GenBank accession numbers are provided in brackets beside the name of the organism. The species considered in this study are highlighted in blue (*Chlamydomonas priscui*), green (*Chlamydomonas malina*) and mauve (*Chlamydomonas klinobasis*).
**Figure S2:** Phylogenetic tree of 111 18S *rRNA* nucleotide sequences inferred from using maximum likelihood analysis is shown to scale (substitutions per site), with branch values representing bootstrap support. GenBank accession numbers are provided in brackets beside the name of the organism. The species considered in this study are highlighted in blue (*Chlamydomonas priscui*), green (*Chlamydomonas malina*) and mauve (*Chlamydomonas klinobasis*).
**Figure S3:** Optimizing the growth and stress conditions in *C. klinobasis* and *C. malina* . (a) Optical density measured at a range of salinities (0.43–70 mM NaCl) in C. *klinobasis* cultures. (b) Optical densities measured at a range of salinities (10–600 mM) in *C. malina* cultures. In both (a) and (b), algal cultures cultured at 4°C. (c) Kinetics of cell death in C. *klinobasis* exposed to non‐permissive temperatures (21°C, 22°C, 23°C). (d) Kinetics of cell death in *C. malina* exposed to non‐permissive temperatures (22°C, 23°C, 24°C). In both (c) and (d), the algae were acclimated to the lowest salinity that supports robust growth (0.43 mM and 30 mM NaCl for C. *klinobasis* and *C. malina* , respectively). The blue line corresponds to the kinetics of cell death in C. *priscui* acclimated to 10 mM NaCl and exposed to 24°C. In all cases, algae were cultured at a constant light intensity of 100 μmol m^−2^ s^−1^. Data are means ± SD of at least three independent experiments.
**Table S1:** A comparison of the growth rates in *C. priscui* (P), *C. malina* (M), and *C. klinobasis* (K) cultured at 4°C and acclimated to high salinity (HS), low salinity (LS), high light (HL) or low light (LL). Data are means ± SD of at least three biological replicates. The lack of statistical difference (*p* > 0.05) between the growth rates of the three species was confirmed with a one‐way ANOVA with a Tukey's post hoc test preformed within each treatment.
**Table S2:** A statistical summary of two‐way ANOVA analyzes for the interaction effects between the experimental variables (salinity, light intensity) and the measured morphological and physiological parameters in *C. priscui*, *C. malina* and *C. klinobasis*. The numbers represent *p*‐values, and significant interactions (*p* < 0.05) are bolded.
**Table S3:** A statistical summary of three‐way ANOVA analyzes for the interaction effects between the experimental variables (salinity, light intensity, time at non‐permissive temperature) and the measured viability and physiological parameters in C. *priscui*, *C. malina* and C. *klinobasis*. The numbers represent *p*‐values, and significant interactions (*p* < 0.05) are bolded.
**Table S4:** A statistical summary of three‐way ANOVA analyzes followed by Dunnett's post hoc test for the energy partitioning parameters measured in C. *prisui*, *C. malina* and C. *klinobasis*. The total absorbed light is distributed between photochemical yield of PSII Y(II), non‐photochemical quenching Y(NPQ), and non‐regulated dissipation Y(NO). In all cases, algae were cultured at 4°C and acclimated to high salinity (HS), low salinity (LS), high light (HL) or low light (LL). Temperature stress was initiated at the mid‐exponential growth phase (OD750 = 0.3–0.4) and corresponds to *t* = 0 h. Data are means ± SD of at least three biological replicates. Statistical significance is indicated by an asterisk and bolded (* = < 0.05, ** = < 0.01, *** = < 0.001, **** = < 0.0001).

## Data Availability

All data is available from the corresponding author on request. Sequences for RbcL from C. malina (PZ227531) and C. klinobasis (PZ227532) are deposited in GenBank.

## References

[ppl71149-bib-0001] Ahmed, F. , E. Leu , A. R. Juhl , et al. 2025. “A Pan‐Arctic Perspective on the Influence of Ice Algae on Sea‐Ice Nutrient Concentrations.” Elementa: Science of the Anthropocene 13: 00059.

[ppl71149-bib-0002] Allakhverdiev, S. I. , V. D. Kreslavski , V. V. Klimov , D. A. Los , R. Carpentier , and P. Mohanty . 2008. “Heat Stress: An Overview of Molecular Responses in Photosynthesis.” Photosynthesis Research 98: 541–550. 10.1007/s11120-008-9331-0 18649006

[ppl71149-bib-0003] Andersen, H. D. , C. Wang , L. Arleth , G. H. Peters , and P. Westh . 2011. “Reconciliation of Opposing Views on Membrane–Sugar Interactions.” Proceedings of the National Academy of Sciences 108: 1874–1878. 10.1073/pnas.1012516108 PMC3033295 21245346

[ppl71149-bib-0004] Anderson, L. G. , and R. W. Macdonald . 2015. “Observing the Arctic Ocean Carbon Cycle in a Changing Environment.” Polar Research 34: 26891.

[ppl71149-bib-0005] Arrigo, K. R. , D. K. Perovich , R. S. Pickart , et al. 2012. “Massive Phytoplankton Blooms Under Arctic Sea Ice.” Science 336: 1408. 10.1126/science.1215065 22678359

[ppl71149-bib-0006] Balzano, S. , P. Gourvil , R. Siano , et al. 2012. “Diversity of Cultured Photosynthetic Flagellates in the Northeast Pacific and Arctic Oceans in Summer.” Biogeosciences 9: 4553–4571.

[ppl71149-bib-0007] Barati, B. , S.‐Y. Gan , P.‐E. Lim , J. Beardall , and S.‐M. Phang . 2019. “Green Algal Molecular Responses to Temperature Stress.” Acta Physiologiae Plantarum 41: 26.

[ppl71149-bib-0008] Bax, N. , C. J. Sands , B. Gogarty , et al. 2021. “Perspective: Increasing Blue Carbon Around Antarctica Is an Ecosystem Service of Considerable Societal and Economic Value Worth Protecting.” Global Change Biology 27: 5–12. 10.1111/gcb.15392 33064891

[ppl71149-bib-0009] Bers, A. V. , F. Momo , I. R. Schloss , and D. Abele . 2013. “Analysis of Trends and Sudden Changes in Long‐Term Environmental Data From King George Island (Antarctica): Relationships Between Global Climatic Oscillations and Local System Response.” Climatic Change 116: 789–803.

[ppl71149-bib-0010] Blanchard‐Wrigglesworth, E. , T. Cox , Z. I. Espinosa , and A. Donohoe . 2023. “The Largest Ever Recorded Heatwave—Characteristics and Attribution of the Antarctic Heatwave of March 2022.” Geophysical Research Letters 50: e2023GL104910.

[ppl71149-bib-0011] Bronselaer, B. , M. Winton , S. M. Griffies , et al. 2018. “Change in Future Climate due to Antarctic Meltwater.” Nature 564: 53–58. 10.1038/s41586-018-0712-z 30455421

[ppl71149-bib-0012] Bryant, C. , R. J. Harris , N. Brothers , et al. 2024. “Cross‐Tolerance: Salinity Gradients and Dehydration Increase Photosynthetic Heat Tolerance in Mangrove Leaves.” Functional Ecology 38: 897–909.

[ppl71149-bib-0013] Buchheim, M. A. , E. A. Michalopulos , and J. A. Buchheim . 2001. “Phylogeny of the Chlorophyceae With Special Reference to the Sphaeropleales: A Study of 18S and 26S rDNA Data.” Journal of Phycology 37: 819–835.

[ppl71149-bib-0014] Cárdenas, C. A. , M. González‐Aravena , and P. A. Santibañez . 2018. “The Importance of Local Settings: Within‐Year Variability in Seawater Temperature at South Bay, Western Antarctic Peninsula.” PeerJ 6: e4289. 10.7717/peerj.4289 PMC5776021 29372123

[ppl71149-bib-0015] Castellani, G. , G. Veyssière , M. Karcher , et al. 2022. “Shine a Light: Under‐Ice Light and Its Ecological Implications in a Changing Arctic Ocean.” Ambio 51: 307–317. 10.1007/s13280-021-01662-3 PMC8692516 34822117

[ppl71149-bib-0016] Castendyk, D. N. , M. K. Obryk , S. Z. Leidman , M. Gooseff , and I. Hawes . 2016. “Lake Vanda: A Sentinel for Climate Change in the McMurdo Sound Region of Antarctica.” Global and Planetary Change 144: 213–227.

[ppl71149-bib-0017] Cheloni, G. , and V. I. Slaveykova . 2021. “Morphological Plasticity in *Chlamydomonas reinhardtii* and Acclimation to Micropollutant Stress.” Aquatic Toxicology 231: 105711. 10.1016/j.aquatox.2020.105711 33338702

[ppl71149-bib-0018] Chen, S.‐T. , N.‐Y. He , J.‐H. Chen , and F.‐Q. Guo . 2017. “Identification of Core Subunits of Photosystem II as Action Sites of HSP21, Which Is Activated by the GUN5‐Mediated Retrograde Pathway in Arabidopsis.” Plant Journal 89: 1106–1118. 10.1111/tpj.13447 27943531

[ppl71149-bib-0019] Clark, P. U. , A. S. Dyke , J. D. Shakun , et al. 2009. “The Last Glacial Maximum.” Science 325: 710–714. 10.1126/science.1172873 19661421

[ppl71149-bib-0020] Constable, A. J. 2022. “Imperatives for Integrated Science and Policy in Managing Greenhouse Gas Risks to the Southern Polar Region.” Global Change Biology 28: 4489–4492. 10.1111/gcb.16219 35575103

[ppl71149-bib-0021] Cook, G. , A. Teufel , I. Kalra , et al. 2019. “The Antarctic Psychrophiles Chlamydomonas spp. UWO241 and ICE‐MDV Exhibit Differential Restructuring of Photosystem I in Response to Iron.” Photosynthesis Research 141: 209–228. 10.1007/s11120-019-00621-0 30729447

[ppl71149-bib-0022] Criscuolo, A. , and S. Gribaldo . 2010. “BMGE (Block Mapping and Gathering With Entropy): A New Software for Selection of Phylogenetic Informative Regions From Multiple Sequence Alignments.” BMC Evolutionary Biology 10: 210. 10.1186/1471-2148-10-210 PMC3017758 20626897

[ppl71149-bib-0023] Cvetkovska, M. , N. P. A. Hüner , and D. R. Smith . 2017. “Chilling Out: The Evolution and Diversification of Psychrophilic Algae With a Focus on Chlamydomonadales.” Polar Biology 40: 1169–1184.

[ppl71149-bib-0024] Cvetkovska, M. , S. Orgnero , N. P. A. Hüner , and D. R. Smith . 2019. “The Enigmatic Loss of Light‐Independent Chlorophyll Biosynthesis From an Antarctic Green Alga in a Light‐Limited Environment.” New Phytologist 222: 651–656. 10.1111/nph.15623 30506801

[ppl71149-bib-0025] Cvetkovska, M. , G. Vakulenko , D. R. Smith , X. Zhang , and N. P. A. Hüner . 2022. “Temperature Stress in Psychrophilic Green Microalgae: Minireview.” Physiologia Plantarum 174: e13811. 10.1111/ppl.13811 36309822

[ppl71149-bib-0026] Cvetkovska, M. , X. Zhang , G. Vakulenko , et al. 2022. “A Constitutive Stress Response Is a Result of Low Temperature Growth in the Antarctic Green Alga *Chlamydomonas* sp. uwo241 .” Plant, Cell & Environment 45: 156–177. 10.1111/pce.14203 34664276

[ppl71149-bib-0027] Dalpadado, P. , K. R. Arrigo , G. L. van Dijken , et al. 2020. “Climate Effects on Temporal and Spatial Dynamics of Phytoplankton and Zooplankton in the Barents Sea.” Progress in Oceanography 185: 102320.

[ppl71149-bib-0028] Darling, K. F. , C. M. Wade , I. A. Stewart , D. Kroon , R. Dingle , and A. J. L. Brown . 2000. “Molecular Evidence for Genetic Mixing of Arctic and Antarctic Subpolar Populations of Planktonic Foraminifers.” Nature 405: 43–47. 10.1038/35011002 10811211

[ppl71149-bib-0029] Davey, M. P. , L. Norman , P. Sterk , et al. 2019. “Snow Algae Communities in Antarctica: Metabolic and Taxonomic Composition.” New Phytologist 222: 1242–1255. 10.1111/nph.15701 PMC6492300 30667072

[ppl71149-bib-0030] Del Castillo, C. E. , S. Signorini , E. M. Karaköylü , and S. Rivero‐Calle . 2019. “Is the Southern Ocean Getting Greener?” Geophysical Research Letters 46: 6034–6040. 10.1029/2019GL083163 PMC7837506 33505102

[ppl71149-bib-0031] Demchenko, E. , T. Mikhailyuk , A. W. Coleman , and T. Pröschold . 2012. “Generic and Species Concepts in Microglena (Previously the *Chlamydomonas monadina* Group) Revised Using an Integrative Approach.” European Journal of Phycology 47: 264–290.

[ppl71149-bib-0032] Dorrell, R. G. , A. Kuo , Z. Füssy , et al. 2023. “Convergent Evolution and Horizontal Gene Transfer in Arctic Ocean Microalgae.” Life Science Alliance 6: e202201833. 10.26508/lsa.202201833 PMC9756366 36522135

[ppl71149-bib-0033] Duncan, R. J. , D. Nielsen , J. E. Søreide , et al. 2024. “Biomolecular Profiles of Arctic Sea‐Ice Diatoms Highlight the Role of Under‐Ice Light in Cellular Energy Allocation.” ISME Communications 4: ycad010. 10.1093/ismeco/ycad010 PMC10848308 38328449

[ppl71149-bib-0034] Duncan, R. J. , J. E. Søreide , Ø. Varpe , et al. 2024. “Spatio‐Temporal Dynamics in Microalgal Communities in Arctic Land‐Fast Sea Ice.” Progress in Oceanography 224: 103248.

[ppl71149-bib-0035] Durack, P. J. , and S. E. Wijffels . 2010. “Fifty‐Year Trends in Global Ocean Salinities and Their Relationship to Broad‐Scale Warming.” Journal of Climate 23, no. 16: 4342–4362. 10.1175/2010JCLI3377.1.

[ppl71149-bib-0036] Eddie, B. , C. Krembs , and S. Neuer . 2008. “Characterization and Growth Response to Temperature and Salinity of Psychrophilic, Halotolerant Chlamydomonas sp. ARC Isolated From Chukchi Sea Ice.” Marine Ecology Progress Series 354: 107–117.

[ppl71149-bib-0037] Feron, S. , R. R. Cordero , A. Damiani , A. Malhotra , G. Seckmeyer , and P. Llanillo . 2021. “Warming Events Projected to Become More Frequent and Last Longer Across Antarctica.” Scientific Reports 11: 19564. 10.1038/s41598-021-98619-z PMC8486840 34599225

[ppl71149-bib-0038] Fox, J. , and S. Weisberg . 2019. An R Companion to Applied Regression. Third ed. SAGE Publications.

[ppl71149-bib-0039] Gegner, H. M. , M. Ziegler , N. Rädecker , C. Buitrago‐López , M. Aranda , and C. R. Voolstra . 2017. “High Salinity Conveys Thermotolerance in the Coral Model Aiptasia.” Biol Open 6: 1943–1948. 10.1242/bio.028878 PMC5769654 29175860

[ppl71149-bib-0040] Gérikas Ribeiro, C. , A. L. dos Santos , P. Gourvil , et al. 2020. “Culturable Diversity of Arctic Phytoplankton During Pack Ice Melting.” Elementa: Science of the Anthropocene 8: 6.

[ppl71149-bib-0041] Gollan, P. J. , and E.‐M. Aro . 2020. “Photosynthetic Signalling During High Light Stress and Recovery: Targets and Dynamics.” Philosophical Transactions of the Royal Society of London. Series B, Biological Sciences 375: 20190406. 10.1098/rstb.2019.0406 PMC7209949 32362249

[ppl71149-bib-0042] Gray, A. , M. Krolikowski , P. Fretwell , et al. 2020. “Remote Sensing Reveals Antarctic Green Snow Algae as Important Terrestrial Carbon Sink.” Nature Communications 11: 2527. 10.1038/s41467-020-16018-w PMC7239900 32433543

[ppl71149-bib-0043] Guindon, S. , J.‐F. Dufayard , V. Lefort , M. Anisimova , W. Hordijk , and O. Gascuel . 2010. “New Algorithms and Methods to Estimate Maximum‐Likelihood Phylogenies: Assessing the Performance of PhyML 3.0.” Systematic Biology 59: 307–321. 10.1093/sysbio/syq010 20525638

[ppl71149-bib-0044] Guo, M. , X.‐S. Wang , H.‐D. Guo , et al. 2022. “Tomato Salt Tolerance Mechanisms and Their Potential Applications for Fighting Salinity: A Review.” Frontiers in Plant Science 13: 949541. 10.3389/fpls.2022.949541 PMC9515470 36186008

[ppl71149-bib-0045] Hadi, S. I. I. A. , H. Santana , P. P. M. Brunale , et al. 2016. “DNA Barcoding Green Microalgae Isolated From Neotropical Inland Waters.” PLoS One 11: e0149284. 10.1371/journal.pone.0149284 PMC4767179 26900844

[ppl71149-bib-0046] Henley, W. J. , K. M. Major , and J. L. Hironaka . 2002. “Response to Salinity and Heat Stress in Two Halotolerant Chlorophyte Algae1.” Journal of Phycology 38: 757–766.

[ppl71149-bib-0047] Hill, V. J. , B. Light , M. Steele , and R. C. Zimmerman . 2018. “Light Availability and Phytoplankton Growth Beneath Arctic Sea Ice: Integrating Observations and Modeling.” Journal of Geophysical Research: Oceans 123: 3651–3667.

[ppl71149-bib-0048] Hodač, L. , C. Hallmann , K. Spitzer , et al. 2016. “Widespread Green Algae Chlorella and Stichococcus Exhibit Polar‐Temperate and Tropical‐Temperate Biogeography.” FEMS Microbiology Ecology 92: fiw122. 10.1093/femsec/fiw122 27279416

[ppl71149-bib-0049] Hoham, R. W. , and D. Remias . 2020. “Snow and Glacial Algae: A Review1.” Journal of Phycology 56: 264–282. 10.1111/jpy.12952 PMC7232433 31825096

[ppl71149-bib-0050] Hu, X. , Y. Yang , F. Gong , et al. 2015. “Protein sHSP26 Improves Chloroplast Performance Under Heat Stress by Interacting With Specific Chloroplast Proteins in Maize ( *Zea mays* ).” Journal of Proteomics 115: 81–92. 10.1016/j.jprot.2014.12.009 25540934

[ppl71149-bib-0051] Huang, B. , Z. Wang , X. Yin , et al. 2021. “Prolonged Marine Heatwaves in the Arctic: 1982–2020.” Geophysical Research Letters 48: e2021GL095590.

[ppl71149-bib-0052] Hui, C. , S. Schmollinger , and A. G. Glaesener . 2023. “Chapter 11–Growth Techniques.” In The Chlamydomonas Sourcebook (Third Edition), edited by U. Goodenough , 287–314. Academic Press.

[ppl71149-bib-0053] Hulatt, C. J. , O. Berecz , E. S. Egeland , R. H. Wijffels , and V. Kiron . 2017. “Polar Snow Algae as a Valuable Source of Lipids?” Bioresource Technology 235: 338–347. 10.1016/j.biortech.2017.03.130 28384586

[ppl71149-bib-0054] Hüner, N. P. A. , A. G. Ivanov , B. Szyszka‐Mroz , L. V. Savitch , D. R. Smith , and V. Kata . 2024. “Photostasis and Photosynthetic Adaptation to Polar Life.” Photosynthesis Research 161: 51–64. 10.1007/s11120-024-01104-7 38865029

[ppl71149-bib-0055] Hüner, N. P. A. , D. R. Smith , M. Cvetkovska , et al. 2022. “Photosynthetic Adaptation to Polar Life: Energy Balance, Photoprotection and Genetic Redundancy.” Journal of Plant Physiology 268: 153557. 10.1016/j.jplph.2021.153557 34922115

[ppl71149-bib-0056] Hüner, N. P. A. , B. Szyszka‐Mroz , A. G. Ivanov , V. Kata , H. Lye , and D. R. Smith . 2023. “Photosynthetic Adaptation and Multicellularity in the Antarctic Psychrophile, *Chlamydomonas priscuii* .” Algal Research 74: 103220.

[ppl71149-bib-0057] Jeffrey, S. W. , and G. F. Humphrey . 1975. “New Spectrophotometric Equations for Determining Chlorophylls *a*, *b*, *c*1 and *c*2 in Higher Plants, Algae and Natural Phytoplankton.” Biochemie und Physiologie der Pflanzen 167: 191–194.

[ppl71149-bib-0058] Ji, X. , J. Cheng , D. Gong , et al. 2018. “The Effect of NaCl Stress on Photosynthetic Efficiency and Lipid Production in Freshwater Microalga— *Scenedesmus obliquus* XJ002.” Science of the Total Environment 633: 593–599. 10.1016/j.scitotenv.2018.03.240 29587228

[ppl71149-bib-0059] Jung, W. , E. J. Kim , S. Lim , et al. 2016. “Cellular Growth and Fatty Acid Content of Arctic Chlamydomonadalean.” Algae 31: 61–71.

[ppl71149-bib-0060] Kalra, I. , X. Wang , M. Cvetkovska , et al. 2020. “Chlamydomonas sp. UWO 241 Exhibits High Cyclic Electron Flow and Rewired Metabolism Under High Salinity.” Plant Physiology 183: 601. 10.1104/pp.19.01280 PMC7271785 32229607

[ppl71149-bib-0061] Kalra, I. , X. Wang , R. Zhang , and R. Morgan‐Kiss . 2023. “High Salt‐Induced PSI‐Supercomplex Is Associated With High CEF and Attenuation of State Transitions.” Photosynthesis Research 157: 65–84. 10.1007/s11120-023-01032-y PMC10484818 37347385

[ppl71149-bib-0062] Karsten, U. , and A. Holzinger . 2014. “Green Algae in Alpine Biological Soil Crust Communities: Acclimation Strategies Against Ultraviolet Radiation and Dehydration.” Biodiversity and Conservation 23: 1845–1858. 10.1007/s10531-014-0653-2 PMC4058318 24954980

[ppl71149-bib-0063] Kassambara, A. 2025. “rstatix: Pipe‐Friendly Framework for Basic Statistical Tests (R v0.7.3).”

[ppl71149-bib-0064] Katoh, K. , and D. M. Standley . 2013. “MAFFT Multiple Sequence Alignment Software Version 7: Improvements in Performance and Usability.” Molecular Biology and Evolution 30: 772–780. 10.1093/molbev/mst010 PMC3603318 23329690

[ppl71149-bib-0065] Khona, D. K. , S. M. Shirolikar , K. K. Gawde , E. Hom , M. A. Deodhar , and J. S. D'Souza . 2016. “Characterization of Salt Stress‐Induced Palmelloids in the Green Alga, *Chlamydomonas reinhardtii* .” Algal Research 16: 434–448.

[ppl71149-bib-0066] Kramer, D. M. , G. Johnson , O. Kiirats , and G. E. Edwards . 2004. “New Fluorescence Parameters for the Determination of QA Redox State and Excitation Energy Fluxes.” Photosynthesis Research 79: 209–218. 10.1023/B:PRES.0000015391.99477.0d 16228395

[ppl71149-bib-0067] Lee, K.‐K. , P.‐E. Lim , S.‐W. Poong , C.‐Y. Wong , S.‐M. Phang , and J. Beardall . 2018. “Growth and Photosynthesis of Chlorella Strains From Polar, Temperate and Tropical Freshwater Environments Under Temperature Stress.” Journal of Oceanology and Limnology 36: 1266–1279.

[ppl71149-bib-0068] Lefort, V. , J.‐E. Longueville , and O. Gascuel . 2017. “SMS: Smart Model Selection in PhyML.” Molecular Biology and Evolution 34: 2422–2424. 10.1093/molbev/msx149 PMC5850602 28472384

[ppl71149-bib-0069] Lehnherr, I. , V. L. St. Louis , M. Sharp , et al. 2018. “The World's Largest High Arctic Lake Responds Rapidly to Climate Warming.” Nature Communications 9: 1290. 10.1038/s41467-018-03685-z PMC5876346 29599477

[ppl71149-bib-0070] Leliaert, F. , D. R. Smith , H. Moreau , et al. 2012. “Phylogeny and Molecular Evolution of the Green Algae.” Critical Reviews in Plant Sciences 31: 1–46.

[ppl71149-bib-0071] Lemieux, C. , A. T. Vincent , A. Labarre , C. Otis , and M. Turmel . 2015. “Chloroplast Phylogenomic Analysis of Chlorophyte Green Algae Identifies a Novel Lineage Sister to the Sphaeropleales (Chlorophyceae).” BMC Evolutionary Biology 15: 264. 10.1186/s12862-015-0544-5 PMC4665822 26620802

[ppl71149-bib-0072] Lemoine, F. , D. Correia , V. Lefort , et al. 2019. “New Generation Phylogenetic Services for Non‐Specialists.” Nucleic Acids Research 47: W260–W265. 10.1093/nar/gkz303 PMC6602494 31028399

[ppl71149-bib-0073] Lenth, R. V. , and J. Piaskowski . 2026. “Emmeans: Estimated Marginal Means, Aka Least‐Squares Means.”

[ppl71149-bib-0074] Letunic, I. , and P. Bork . 2021. “Interactive Tree of Life (iTOL) v5: An Online Tool for Phylogenetic Tree Display and Annotation.” Nucleic Acids Research 49: W293–W296. 10.1093/nar/gkab301 PMC8265157 33885785

[ppl71149-bib-0075] Li, W. , J. Dolhi‐Binder , Z. Cariani , and R. Morgan‐Kiss . 2019. “Drivers of Protistan Community Autotrophy and Heterotrophy in Chemically Stratified Antarctic Lakes.” Aquatic Microbial Ecology 82: 225–239.

[ppl71149-bib-0076] Liska, A. J. , A. Shevchenko , U. Pick , and A. Katz . 2004. “Enhanced Photosynthesis and Redox Energy Production Contribute to Salinity Tolerance in Dunaliella as Revealed by Homology‐Based Proteomics.” Plant Physiology 136: 2806–2817. 10.1104/pp.104.039438 PMC523343 15333751

[ppl71149-bib-0077] Liu, C. , X. Huang , X. Wang , X. Zhang , and G. Li . 2006. “Phylogenetic Studies on Two Strains of Antarctic Ice Algae Based on Morphological and Molecular Characteristics.” Phycologia 45: 190–198.

[ppl71149-bib-0078] Lodeyro, A. F. , A. R. Krapp , and N. Carrillo . 2021. “Photosynthesis and Chloroplast Redox Signaling in the Age of Global Warming: Stress Tolerance, Acclimation, and Developmental Plasticity.” Journal of Experimental Botany 72: 5919–5937. 10.1093/jxb/erab270 34111246

[ppl71149-bib-0079] Lurling, M. , and W. Beekman . 2006. “Palmelloids Formation in *Chlamydomonas reinhardtii* : Defence Against Rotifer Predators?” Annales de Limnologie ‐ International Journal of Limnology 42: 65–72.

[ppl71149-bib-0080] Malapascua, J. , C. Jerez , M. Sergejevová , F. Figueroa , and J. Masojídek . 2014. “Photosynthesis Monitoring to Optimize Growth of Microalgal Mass Cultures: Application of Chlorophyll Fluorescence Techniques.” Aquatic Biology 22: 123–140.

[ppl71149-bib-0081] Manizza, M. , D. Carroll , D. Menemenlis , H. Zhang , and C. E. Miller . 2023. “Modeling the Recent Changes of Phytoplankton Blooms Dynamics in the Arctic Ocean.” Journal of Geophysical Research: Oceans 128: e2022JC019152.

[ppl71149-bib-0082] Meiners, K. M. , D. Lannuzel , E. Leu , and K. A. Brown . 2025. “Nutrients and Organic Matter Dynamics in Sea Ice.” In Sea Ice, 575–622. John Wiley & Sons, Ltd.

[ppl71149-bib-0083] Morales‐Sánchez, D. , P. S. C. Schulze , V. Kiron , and R. H. Wijffels . 2020a. “Temperature‐Dependent Lipid Accumulation in the Polar Marine Microalga Chlamydomonas Malina RCC2488.” Frontiers in Plant Science 11: 619064. 10.3389/fpls.2020.619064 PMC7785989 33424911

[ppl71149-bib-0084] Morales‐Sánchez, D. , P. S. C. Schulze , V. Kiron , and R. H. Wijffels . 2020b. “Production of Carbohydrates, Lipids and Polyunsaturated Fatty Acids (PUFA) by the Polar Marine Microalga *Chlamydomonas malina* RCC2488.” Algal Research 50: 102016.

[ppl71149-bib-0085] Morgan‐Kiss, R. M. , and M. D. Guiry . 2024. “Validation of “*Chlamydomonas priscui*” (“*Chlamydomonas* sp. UWO241” Chlamydomonadaceae, Chlorophyceae), a Psychrophilic Organism From the Antarctic Widely Used in Research on Cold Adaptation of Photosynthesis.” Notulae Algarum 346: 1–2.

[ppl71149-bib-0086] Morgan‐Kiss, R. M. , D. Popson , R. Pereira , et al. 2024. “Sentinel Protist Taxa of the McMurdo Dry Valley Lakes, Antarctica: A Review.” Frontiers in Ecology and Evolution 12: 1323472.

[ppl71149-bib-0087] Morita, R. Y. 1975. “Psychrophilic Bacteria.” Bacteriological Reviews 39: 144–167. 10.1128/br.39.2.144-167.1975 PMC413900 1095004

[ppl71149-bib-0088] Myers‐Smith, I. H. , J. T. Kerby , G. K. Phoenix , et al. 2020. “Complexity Revealed in the Greening of the Arctic.” Nature Climate Change 10: 106–117. 10.1038/s41558-019-0688-1.

[ppl71149-bib-0089] Nakada, T. , K. Misawa , and H. Nozaki . 2008. “Molecular Systematics of Volvocales (Chlorophyceae, Chlorophyta) Based on Exhaustive 18S *r*RNA Phylogenetic Analyses.” Molecular Phylogenetics and Evolution 48: 281–291. 10.1016/j.ympev.2008.03.016 18430591

[ppl71149-bib-0090] Nakada, T. , M. Tomita , J.‐T. Wu , and H. Nozaki . 2016. “Taxonomic Revision of Chlamydomonas Subg. Amphichloris (Volvocales, Chlorophyceae), With Resurrection of the Genus Dangeardinia and Descriptions of Ixipapillifera Gen. Nov. and Rhysamphichloris Gen. Nov.” Journal of Phycology 52: 283–304. 10.1111/jpy.12397 27037593

[ppl71149-bib-0091] Neale, P. J. , and J. C. Priscu . 1995. “The Photosynthetic Apparatus of Phytoplankton From a Perennially Ice‐Covered Antarctic Lake: Acclimation to an Extreme Shade Environment.” Plant and Cell Physiology 36: 253–263.

[ppl71149-bib-0092] Ng, L. M. , S. Komaki , H. Takahashi , T. Yamano , H. Fukuzawa , and T. Hashimoto . 2022. “Hyperosmotic Stress‐Induced Microtubule Disassembly in *Chlamydomonas reinhardtii* .” BMC Plant Biology 22: 46. 10.1186/s12870-022-03439-6 PMC8783414 35065609

[ppl71149-bib-0093] Nievola, C. C. , C. P. Carvalho , V. Carvalho , and E. Rodrigues . 2017. “Rapid Responses of Plants to Temperature Changes.” Temperature 4: 371–405. 10.1080/23328940.2017.1377812 PMC5800372 29435478

[ppl71149-bib-0094] Obryk, M. K. , P. T. Doran , and J. C. Priscu . 2019. “Prediction of Ice‐Free Conditions for a Perennially Ice‐Covered Antarctic Lake.” Journal of Geophysical Research ‐ Earth Surface 124: 686–694.

[ppl71149-bib-0095] Pascual, L. S. , D. Mohanty , R. Sinha , et al. 2025. “Enhanced Cell Aggregation in the *Chlamydomonas reinhardtii* rbo1 Mutant in Response to Multifactorial Stress Combination.” Plant Physiology 199: kiaf551. 10.1093/plphys/kiaf551 41206142

[ppl71149-bib-0096] Patriarche, J. D. , J. C. Priscu , C. Takacs‐Vesbach , et al. 2021. “Year‐Round and Long‐Term Phytoplankton Dynamics in Lake Bonney, a Permanently Ice‐Covered Antarctic Lake.” Journal of Geophysical Research: Biogeosciences 126: e2020JG005925.

[ppl71149-bib-0097] Pocock, T. , M.‐A. Lachance , T. Pröschold , J. C. Priscu , S. S. Kim , and N. P. A. Huner . 2004. “Identification of A Psychrophilic Green Alga From Lake Bonney Antarctica: *Chlamydomonas RAUDENSIS* ETTL. (UWO 241) *Chlorophyceae* .” Journal of Phycology 40: 1138–1148.

[ppl71149-bib-0098] Pocock, T. , A. Vetterli , and S. Falk . 2011. “Evidence for Phenotypic Plasticity in the Antarctic Extremophile Chlamydomonas Raudensis Ettl. UWO 241.” Journal of Experimental Botany 62: 1169–1177. 10.1093/jxb/erq347 PMC3022403 21041369

[ppl71149-bib-0099] Poirier, M. C. , K. Fugard , and M. Cvetkovska . 2025. “Light Quality Affects Chlorophyll Biosynthesis and Photosynthetic Performance in Antarctic Chlamydomonas.” Photosynthesis Research 163: 9. 10.1007/s11120-024-01127-0 39832016

[ppl71149-bib-0100] Popson, D. , S. D'Silva , K. Wheeless , and R. Morgan‐Kiss . 2024. “Permanent Stress Adaptation and Unexpected High Light Tolerance in the Shade‐Adapted Chlamydomonas Priscui.” Plants 13: 2254. 10.3390/plants13162254 PMC11359158 39204690

[ppl71149-bib-0101] Posch, B. C. , H. Amoanimaa‐Dede , L. M. T. Aparecido , et al. 2026. “High‐Temperature Acclimation of Photosystem II in Land Plants.” New Phytologist 249: 1108–1123. 10.1111/nph.70661 41178112

[ppl71149-bib-0102] Possmayer, M. , G. Berardi , B. F. N. Beall , C. G. Trick , N. P. A. Hüner , and D. P. Maxwell . 2011. “Plasticity of the Psychrophilic Green Alga Chlamydomonas Raudensis (UWO 241) (Chlorophyta) to Supraoptimal Temperature STRESS1: Heat Stress in A Psychrophilic Green Alga.” Journal of Phycology 47: 1098–1109. 10.1111/j.1529-8817.2011.01047.x 27020192

[ppl71149-bib-0103] Possmayer, M. , R. K. Gupta , B. Szyszka‐Mroz , et al. 2016. “Resolving the Phylogenetic Relationship Between *Chlamydomonas* sp. uwo 241 and *Chlamydomonas raudensis* Sag 49.72 (Chlorophyceae) With Nuclear and Plastid dna Sequences.” Journal of Phycology 52: 305–310. 10.1111/jpy.12383 PMC5067557 27037594

[ppl71149-bib-0104] Pushpavalli, R. , J. D. Berger , N. C. Turner , K. H. M. Siddique , T. D. Colmer , and V. Vadez . 2020. “Cross‐Tolerance for Drought, Heat and Salinity Stresses in Chickpea ( *Cicer arietinum* L.).” Journal of Agronomy and Crop Science 206: 405–419.

[ppl71149-bib-0105] Rappaport, H. B. , and A. M. Oliverio . 2023. “Extreme Environments Offer an Unprecedented Opportunity to Understand Microbial Eukaryotic Ecology, Evolution, and Genome Biology.” Nature Communications 14: 4959. 10.1038/s41467-023-40657-4 PMC10432404 37587119

[ppl71149-bib-0106] Ratcliff, W. C. , M. D. Herron , K. Howell , J. T. Pentz , F. Rosenzweig , and M. Travisano . 2013. “Experimental Evolution of an Alternating Uni‐ and Multicellular Life Cycle in *Chlamydomonas reinhardtii* .” Nature Communications 4: 2742. 10.1038/ncomms3742 PMC3831279 24193369

[ppl71149-bib-0107] Rheinlænder, J. W. , H. Regan , P. Rampal , G. Boutin , E. Ólason , and R. Davy . 2024. “Breaking the Ice: Exploring the Changing Dynamics of Winter Breakup Events in the Beaufort Sea.” Journal of Geophysical Research: Oceans 129: e2023JC020395.

[ppl71149-bib-0108] Rillig, M. C. , M. Ryo , A. Lehmann , et al. 2019. “The Role of Multiple Global Change Factors in Driving Soil Functions and Microbial Biodiversity.” Science 366: 886–890. 10.1126/science.aay2832 PMC6941939 31727838

[ppl71149-bib-0109] Rivero, R. M. , T. C. Mestre , R. Mittler , F. Rubio , F. Garcia‐Sanchez , and V. Martinez . 2014. “The Combined Effect of Salinity and Heat Reveals a Specific Physiological, Biochemical and Molecular Response in Tomato Plants.” Plant, Cell & Environment 37: 1059–1073. 10.1111/pce.12199 24028172

[ppl71149-bib-0110] Roland, T. P. , O. T. Bartlett , D. J. Charman , et al. 2024. “Sustained Greening of the Antarctic Peninsula Observed From Satellites.” Nature Geoscience 17: 1121–1126.

[ppl71149-bib-0111] Saha, M. , J. Stroeve , D. Isleifson , et al. 2025. “Snow Depth Estimation on Leadless Landfast Ice Using Cryo2Ice Satellite Observations.” Cryosphere 19: 325–346.

[ppl71149-bib-0112] Santos, T. F. , H. Pereira , L. Schüler , et al. 2025. “Enhancement of Heat Tolerance by Salt Stress in Tetraselmis Striata CTP4: Impacts on HSP Gene Expression, Pigments, and Proximal Composition.” Journal of Applied Phycology 37: 287–301.

[ppl71149-bib-0113] Schroda, M. , D. Hemme , and T. Mühlhaus . 2015. “The *Chlamydomonas* Heat Stress Response.” Plant Journal 82: 466–480. 10.1111/tpj.12816 25754362

[ppl71149-bib-0114] Schroda, M. , O. Vallon , F. A. Wollman , and C. F. Beck . 1999. “A Chloroplast‐Targeted Heat Shock Protein 70 (HSP70) Contributes to the Photoprotection and Repair of Photosystem II During and After Photoinhibition.” Plant Cell 11: 1165–1178. 10.1105/tpc.11.6.1165 PMC144243 10368186

[ppl71149-bib-0115] Segawa, T. , R. Matsuzaki , N. Takeuchi , et al. 2018. “Bipolar Dispersal of Red‐Snow Algae.” Nature Communications 9: 3094. 10.1038/s41467-018-05521-w PMC6079020 30082897

[ppl71149-bib-0116] Segawa, T. , T. Yonezawa , R. Matsuzaki , et al. 2023. “Evolution of Snow Algae, From Cosmopolitans to Endemics, Revealed by DNA Analysis of Ancient Ice.” ISME Journal 17: 491–501. 10.1038/s41396-023-01359-3 PMC10030584 36650274

[ppl71149-bib-0117] Shaar‐Moshe, L. , E. Blumwald , and Z. Peleg . 2017. “Unique Physiological and Transcriptional Shifts Under Combinations of Salinity, Drought, and Heat.” Plant Physiology 174: 421–434. 10.1104/pp.17.00030 PMC5411152 28314795

[ppl71149-bib-0118] Sherwell, S. , I. Kalra , W. Li , D. M. McKnight , J. C. Priscu , and R. M. Morgan‐Kiss . 2022. “Antarctic Lake Phytoplankton and Bacteria From Near‐Surface Waters Exhibit High Sensitivity to Climate‐Driven Disturbance.” Environmental Microbiology 24: 6017–6032. 10.1111/1462-2920.16113 PMC10084183 35860854

[ppl71149-bib-0119] Shetty, P. , M. M. Gitau , and G. Maróti . 2019. “Salinity Stress Responses and Adaptation Mechanisms in Eukaryotic Green Microalgae.” Cells 8: 1657. 10.3390/cells8121657 PMC6952985 31861232

[ppl71149-bib-0120] Silvano, A. , S. R. Rintoul , B. Peña‐Molino , et al. 2018. “Freshening by Glacial Meltwater Enhances Melting of Ice Shelves and Reduces Formation of Antarctic Bottom Water.” Science Advances 4: eaap9467. 10.1126/sciadv.aap9467 PMC5906079 29675467

[ppl71149-bib-0121] Slater, T. , I. R. Lawrence , I. N. Otosaka , et al. 2021. “Review Article: Earth's Ice Imbalance.” Cryosphere 15: 233–246.

[ppl71149-bib-0122] Smoot, C. A. , and R. R. Hopcroft . 2017. “Depth‐Stratified Community Structure of Beaufort Sea Slope Zooplankton and Its Relations to Water Masses.” Journal of Plankton Research 39: 79–91.

[ppl71149-bib-0123] Soto, D. F. , I. Gómez , and P. Huovinen . 2023. “Antarctic Snow Algae: Unraveling the Processes Underlying Microbial Community Assembly During Blooms Formation.” Microbiome 11: 200. 10.1186/s40168-023-01643-6 PMC10478455 37667346

[ppl71149-bib-0124] Spolaor, A. , F. Scoto , C. Larose , et al. 2024. “Climate Change Is Rapidly Deteriorating the Climatic Signal in Svalbard Glaciers.” Cryosphere 18: 307–320.

[ppl71149-bib-0125] Stahl‐Rommel, S. , I. Kalra , S. D'Silva , et al. 2021. “Cyclic Electron Flow (CEF) and Ascorbate Pathway Activity Provide Constitutive Photoprotection for the Photopsychrophile, Chlamydomonas sp. UWO 241 (Renamed Chlamydomonas Priscuii).” Photosynthesis Research 151: 235–250. 10.1007/s11120-021-00877-5. 34609708

[ppl71149-bib-0126] Stibal, M. , J. Elster , M. Sabacká , and K. Kastovská . 2007. “Seasonal and Diel Changes in Photosynthetic Activity of the Snow Alga *Chlamydomonas nivalis* (Chlorophyceae) From Svalbard Determined by Pulse Amplitude Modulation Fluorometry.” FEMS Microbiology Ecology 59: 265–273. 10.1111/j.1574-6941.2006.00264.x 17313577

[ppl71149-bib-0127] Stroeve, J. , and D. Notz . 2018. “Changing State of Arctic Sea Ice Across All Seasons.” Environmental Research Letters 13: 103001.

[ppl71149-bib-0128] Sun, Z. , F. X. Cunningham , and E. Gantt . 1998. “Differential Expression of Two Isopentenyl Pyrophosphate Isomerases and Enhanced Carotenoid Accumulation in a Unicellular Chlorophyte.” Proceedings of the National Academy of Sciences of the United States of America 95: 11482–11488. 10.1073/pnas.95.19.11482 PMC21669 9736763

[ppl71149-bib-0129] Suwannachuen, N. , K. Leetanasaksakul , S. Roytrakul , et al. 2023. “Palmelloid Formation and Cell Aggregation Are Essential Mechanisms for High Light Tolerance in a Natural Strain of *Chlamydomonas reinhardtii* .” International Journal of Molecular Sciences 24: 8374. 10.3390/ijms24098374 PMC10179368 37176080

[ppl71149-bib-0130] Suzuki, H. , C. J. Hulatt , R. H. Wijffels , and V. Kiron . 2019. “Growth and LC‐PUFA Production of the Cold‐Adapted Microalga Koliella Antarctica in Photobioreactors.” Journal of Applied Phycology 31: 981–997.

[ppl71149-bib-0131] Szyszka, B. , A. G. Ivanov , and N. P. A. Hüner . 2007. “Psychrophily Is Associated With Differential Energy Partitioning, Photosystem Stoichiometry and Polypeptide Phosphorylation in Chlamydomonas Raudensis.” Biochimica et Biophysica Acta, Bioenergetics 1767: 789–800. 10.1016/j.bbabio.2006.12.001 17234152

[ppl71149-bib-0132] Szyszka‐Mroz, B. , A. G. Ivanov , C. G. Trick , and N. P. A. Hüner . 2022. “Palmelloid Formation in the Antarctic Psychrophile, Chlamydomonas Priscuii, Is Photoprotective.” Frontiers in Plant Science 13: 911035. 10.3389/fpls.2022.911035 PMC9470844 36119589

[ppl71149-bib-0133] Takizawa, K. , S. Takahashi , N. P. A. Hüner , and J. Minagawa . 2009. “Salinity Affects the Photoacclimation of Chlamydomonas Raudensis Ettl UWO241.” Photosynthesis Research 99: 195–203. 10.1007/s11120-008-9397-8 19137412

[ppl71149-bib-0134] Taylor, R. L. , D. M. Semeniuk , C. D. Payne , et al. 2013. “Colimitation by Light, Nitrate, and Iron in the Beaufort Sea in Late Summer.” Journal of Geophysical Research: Oceans 118: 3260–3277.

[ppl71149-bib-0135] Tonboe, R. T. , V. Nandan , J. Yackel , S. Kern , L. T. Pedersen , and J. Stroeve . 2021. “Simulated ka‐ and Ku‐Band Radar Altimeter Height and Freeboard Estimation on Snow‐Covered Arctic Sea Ice.” Cryosphere 15: 1811–1822.

[ppl71149-bib-0136] Tremblay, J.‐É. , D. Robert , D. E. Varela , et al. 2012. “Current State and Trends in Canadian Arctic Marine Ecosystems: I. Primary Production.” Climatic Change 115: 161–178.

[ppl71149-bib-0137] Tremblay, J.‐É. , K. Simpson , J. Martin , et al. 2008. “Vertical Stability and the Annual Dynamics of Nutrients and Chlorophyll Fluorescence in the Coastal, Southeast Beaufort Sea.” Journal of Geophysical Research: Oceans 113: 1–14.

[ppl71149-bib-0138] Trentin, R. , E. Negrisolo , E. Moschin , D. Veronese , M. Cecchetto , and I. Moro . 2022. “Microglena Antarctica sp. Nov. a New Antarctic Green Alga From Inexpressible Island (Terra Nova Bay, Ross Sea) Revealed Through an Integrative Approach.” Diversity 14: 337.

[ppl71149-bib-0139] Turner, J. , H. Lu , J. King , et al. 2021. “Extreme Temperatures in the Antarctic.” Journal of Climate 34, no. 7: 2653–2668. 10.1175/JCLI-D-20-0538.1.

[ppl71149-bib-0140] Urbański, J. A. , and D. Litwicka . 2022. “The Decline of Svalbard Land‐Fast Sea Ice Extent as a Result of Climate Change.” Oceanologia 64: 535–545.

[ppl71149-bib-0141] van Leeuwe, M. A. , L. Tedesco , K. R. Arrigo , et al. 2018. “Microalgal Community Structure and Primary Production in Arctic and Antarctic Sea Ice: A Synthesis.” Elementa: Science of the Anthropocene 6: 4.

[ppl71149-bib-0142] Vineeth, T. V. , G. K. Krishna , P. H. Pandesha , et al. 2023. “Photosynthetic Machinery Under Salinity Stress: Trepidations and Adaptive Mechanisms.” Photosynthetica 61: 73–93. 10.32615/ps.2023.002 PMC11515832 39650121

[ppl71149-bib-0143] Vyverman, W. , E. Verleyen , K. Sabbe , et al. 2007. “Historical Processes Constrain Patterns in Global Diatom Diversity.” Ecology 88: 1924–1931. 10.1890/06-1564.1 17824422

[ppl71149-bib-0144] Williamson, C. J. , J. Cook , A. Tedstone , et al. 2020. “Algal Photophysiology Drives Darkening and Melt of the Greenland Ice Sheet.” Proceedings of the National Academy of Sciences 117: 5694–5705. 10.1073/pnas.1918412117 PMC7084142 32094168

[ppl71149-bib-0145] Wolf, K. K. E. , C. J. M. Hoppe , L. Rehder , E. Schaum , U. John , and B. Rost . 2024. “Heatwave Responses of Arctic Phytoplankton Communities Are Driven by Combined Impacts of Warming and Cooling.” Science Advances 10: eadl5904. 10.1126/sciadv.adl5904 PMC11100554 38758795

[ppl71149-bib-0146] Wunderling, N. , M. Willeit , J. F. Donges , and R. Winkelmann . 2020. “Global Warming due to Loss of Large Ice Masses and Arctic Summer Sea Ice.” Nature Communications 11: 5177. 10.1038/s41467-020-18934-3 PMC7591863 33110092

[ppl71149-bib-0147] Yamori, W. , and T. Shikanai . 2016. “Physiological Functions of Cyclic Electron Transport Around Photosystem I in Sustaining Photosynthesis and Plant Growth.” Annual Review of Plant Biology 67: 81–106. 10.1146/annurev-arplant-043015-112002 26927905

[ppl71149-bib-0148] Young, J. N. , and K. Schmidt . 2020. “It's What's Inside That Matters: Physiological Adaptations of High‐Latitude Marine Microalgae to Environmental Change.” New Phytologist 227: 1307–1318. 10.1111/nph.16648 32391569

[ppl71149-bib-0149] Zahra, N. , M. B. Hafeez , A. Ghaffar , et al. 2023. “Plant Photosynthesis Under Heat Stress: Effects and Management.” Environmental and Experimental Botany 206: 105178.

[ppl71149-bib-0150] Zandalinas, S. I. , and R. Mittler . 2022. “Plant Responses to Multifactorial Stress Combination.” New Phytologist 234: 1161–1167. 10.1111/nph.18087 35278228

[ppl71149-bib-0151] Zandalinas, S. I. , S. Sengupta , F. B. Fritschi , R. K. Azad , R. Nechushtai , and R. Mittler . 2021. “The Impact of Multifactorial Stress Combination on Plant Growth and Survival.” New Phytologist 230: 1034–1048. 10.1111/nph.17232 PMC8048544 33496342

[ppl71149-bib-0152] Zhang, N. , E. M. Mattoon , W. McHargue , et al. 2022. “Systems‐Wide Analysis Revealed Shared and Unique Responses to Moderate and Acute High Temperatures in the Green Alga *Chlamydomonas reinhardtii* .” Communications Biology 5: 460. 10.1038/s42003-022-03359-z PMC9106746 35562408

[ppl71149-bib-0153] Zhang, X. , M. Cvetkovska , R. Morgan‐Kiss , N. P. A. Hüner , and D. R. Smith . 2021. “Draft Genome Sequence of the Antarctic Green Alga Chlamydomonas sp. UWO241.” iScience 24: 102084. 10.1016/j.isci.2021.102084 PMC7887394 33644715

[ppl71149-bib-0154] Zhang, Z. , C. Qu , R. Yao , et al. 2019. “The Parallel Molecular Adaptations to the Antarctic Cold Environment in Two Psychrophilic Green Algae.” Genome Biology and Evolution 11: 1897–1908. 10.1093/gbe/evz104 PMC6628873 31106822

[ppl71149-bib-0155] Zhang, Z. , C. Qu , K. Zhang , et al. 2020. “Adaptation to Extreme Antarctic Environments Revealed by the Genome of a Sea Ice Green Alga.” Current Biology 30: 3330–3341.e7. 10.1016/j.cub.2020.06.029 32619486

[ppl71149-bib-0156] Zhu, L. , A. P. Scafaro , E. Vierling , et al. 2024. “Heat Tolerance of a Tropical–Subtropical Rainforest Tree Species Polyscias Elegans: Time‐Dependent Dynamic Responses of Physiological Thermostability and Biochemistry.” New Phytologist 241: 715–731. 10.1111/nph.19356 37932881

